# Plant-Based Antioxidants in Gluten-Free Bread Production: Sources, Technological and Sensory Aspects, Enhancing Strategies and Constraints

**DOI:** 10.3390/antiox13020142

**Published:** 2024-01-23

**Authors:** Marijana Djordjević, Miljana Djordjević, Małgorzata Starowicz, Urszula Krupa-Kozak

**Affiliations:** 1 Institute of Food Technology in Novi Sad, University of Novi Sad, Blvd. Cara Lazara 1, 21000 Novi Sad, Serbia; miljana.djordjevic@fins.uns.ac.rs; 2 Department of Chemistry and Biodynamics of Food, Institute of Animal Reproduction and Food Research of Polish Academy of Sciences, Tuwima 10 Street, 10-748 Olsztyn, Poland; m.starowicz@pan.olsztyn.pl (M.S.); u.krupa-kozak@pan.olsztyn.pl (U.K.-K.)

**Keywords:** gluten-free bakery products, bioactive compounds, polyphenols, antioxidant activity, quantification methods, sensory properties, bioaccessibility and bioavailability, side-streams valorization

## Abstract

The recognized contribution of antioxidant compounds to overall health maintenance and spotted deficiencies in celiac patients’ diets has driven more intensive research regarding antioxidant compounds’ inclusion in gluten-free bread (GFB) production during the last decade. The presented review gathered information that provided insights into plant-based antioxidant sources which are applicable in GFB production through the resulting changes in the technological, sensory, and nutritional quality of the resulting antioxidant-enriched GFB. The influence of the bread-making process on the antioxidant compounds’ content alteration and applied methods for their quantification in GFB matrices were also discussed, together with strategies for enhancing the antioxidant compounds’ content, their bioaccessibility, and their bioavailability, highlighting the existing contradictions and constraints. The addition of plant-based antioxidant compounds generally improved the antioxidant content and activity of GFB, without a profound detrimental effect on its technological quality and sensory acceptability, and with the extent of the improvement being dependent on the source richness and the amount added. The determination of a pertinent amount and source of plant-based antioxidant material that will result in the production of GFB with desirable nutritional, sensory, and technological quality, as well as biological activity, remains a challenge to be combated by elucidation of the potential mechanism of action and by the standardization of quantification methods for antioxidant compounds.

## 1. Introduction

Consistent research regarding antioxidants in bakery products during the last decades [1,2,3,4,5] has granted additional insight into plant-based antioxidants’ role, going beyond their initially assigned preservation purposes [6,7]. Established health benefits of the antioxidant-rich diet arise from enabled oxidative stress reduction causing a delay in biological aging and lowering the risk of oxidative stress-driven diseases and chronic degenerative diseases such as Alzheimer’s, cancer, liver, and cardiovascular diseases [1,8,9].

A strong association between oxidative stress and celiac disease is established [10] and ascribed to a disruption of the pro-oxidant–antioxidant balance in the small intestinal mucosa as a corollary of free radicals’ overproduction caused by gliadin fragments ingestion [11,12]. This condition is further linked with greater damage of already malfunctioning mucosa in celiac patients. Only a gluten-free (GF) diet rich in antioxidant compounds can aid in halting a damaged mucosa [10,13,14,15]. Nevertheless, the tendency of celiac patients to avoid food that is naturally rich in antioxidant compounds while being free of gluten, like fruits and vegetables, and the often improperly balanced nutritional value and poor quality of GF products are the main obstacles to enhanced antioxidant supply and health maintenance. Another arising concern is the “health halo” effect [16,17,18], representing an enlarged number of healthy individuals who choose a GF diet, risking nutrient deficiency. Concomitantly, the mentioned consumers’ requirements enabled the progressive growth of the GF product market from niche to mainstream [19].

The FDA’s recommendation for antioxidant compound intake is 3000–5000 μmol TE per day (ORAC), whilst an intake greater than 10,000 μmol TE per day (ORAC) is associated with beneficial health outcomes [20,21]. Gluten-free bread (GFB), as an integral part of everyday meals in celiac patient’s diet, can be an adjuvant in combating the aforementioned challenges and recommendations. Furthermore, taking into account strict adherence to a GF diet as a standalone therapy, it could be speculated that antioxidant-enriched GFB can serve as a tool in the therapeutic diet intended for celiac patients. Hence, there are multiple reasons for GFB enhancement with antioxidant compounds:

- To match and/or further enhance the nutritional properties of GFB compared to regular wheat bread;
- To improve the overall quality of the GF diet;
- To assist with healing of the damaged mucosa in celiac patients;
- Contribution to broader health effects.

However, the production of GFB with well-balanced nutritional, technological, and sensory qualities remains a bottleneck requiring further addressing. In this context, GF plant-based raw materials and ingredients that are rich in antioxidant compounds such as GF cereals, pseudocereals, legumes, fruits, and vegetables, as well as their by-products, are key players in completing a nutritionally reinforced GFB.

Antioxidant compounds have become an emerging topic in GFB research, as confirmed by 34 research articles published between 2020 and 2023, compared to the 23 research articles published from 2010 to 2020 whose primary approach was antioxidant analysis in GFB. The antioxidant-rich plant-based raw materials and additives, as well as their quantities, used in diverse GF bakery products were well summarized in the previous review [5]. Still, there are many aspects to be elucidated, including the variety of methods used for antioxidant compounds’ quantification and identification, changes in their content induced by bread-making, their influence on GFB technological and sensory features, and their bioavailability from the GFB matrix, which is crucial for the expression of biological activity. Therefore, this review summarizes antioxidants’ definition, their basic sources, and methods of their quantification and identification, and presents the technological and sensory qualities of antioxidant-enriched GFB, including bioaccessibility and bioavailability studies. Moreover, the existing constraints and enhancing strategies are also highlighted and discussed.

For this review, a literature search was performed in the Scopus database (Elsevier) using the advanced search option. Separate searches were conducted for five query strings which subsequently formed one unique searchable query string. The first string was comprised of the terms “bioactive compounds” and “phenolic compounds”; the second, included sources of phenolic compounds such as “fruits”, “vegetables”, “herbs”, “by-products”, or “waste”; the third enveloped “health benefits” and “antioxidant properties” of the phenolic compounds and similar terms; the fourth and fifth were associated with developed products, i.e., “gluten-free bread” and “flour”, excluding “gluten-free cakes”, “biscuits”, and “cookies”. The setup included a search within the titles, abstracts, and keywords of the research articles published from 2010 to 2023, resulting in 46 hits. Additionally, independent searches were run in the Web of Science database and Google Scholar search engine using the search terms “gluten-free bread” AND “antioxidant activity” and “gluten-free bread” AND “polyphenols” to ensure a more extensive examination of the literature.

## 2. Definition, Classification and Health-Related Effects of Antioxidant Compounds

Molecular oxygen is a crucial chemical element in the oxidative metabolism of aerobes, enabling energy production in the form of ATP, as well as biosynthetic and detoxification reactions in the organism [22]. As a by-product of normal metabolic processes involving oxygen, nitrogen, and sulphur, free radicals are formed. Free radicals present molecular entities or molecular fragments (atoms, ions) that are capable of existing independently and possessing one or more unpaired electrons, which makes them highly unstable and reactive towards other molecules [23]. There are three classes of radical species: the reactive oxygen species (ROS), the reactive nitrogen species (RNS), and the reactive sulphur species (RSS) [24].

Among the mentioned classes, ROS are the most reactive and, besides radicals like superoxide (O_2_•−), hydroxyl (−•OH), peroxyl (ROO•), and alkyloxy radical (RO•), also include non-radical derivatives of oxygen such as hydrogen peroxide (H_2_O_2_), ozone (O_3_), singlet oxygen (^1^O_2_), and hypochlorous acid (HOCl) [25,26]. ROS, together with RNS, have a function in cell signaling, the activation of apoptosis, gene expression, and ion transportation, making them significant actors in the innate immune system [24,27]. However, an overproduction of ROS can trigger an adverse chain reaction in the body, resulting in the damage of proteins, lipids, RNA, and DNA; impairment of cell membranes; and disturbances in cellular processes and normal cell division [27,28]. A high production of ROS is not only a consequence of naturally occurring metabolic processes (endogenous sources), but could also be induced by the impact of various environmental factors (exogenous sources) like ozone radiation, pollution, pesticides, industrial chemicals, and risk behavior factors such as stress, smoking, or excess of physical activity [24,27]. An antioxidant defense system is a mechanism responsible for combatting the excess of free radicals in an organism and maintaining the equilibrium between ROS and antioxidants, thus impairing oxidative stress occurrence. A rise in oxidative stress can be related to different pathological conditions such as ageing, arthritis, asthma, autoimmune diseases, carcinogenesis, cardiovascular dysfunction, cataract, diabetes, neurodegenerative diseases, Alzheimer’s disease, and Parkinson’s dementia [9].

**Figure 1:**
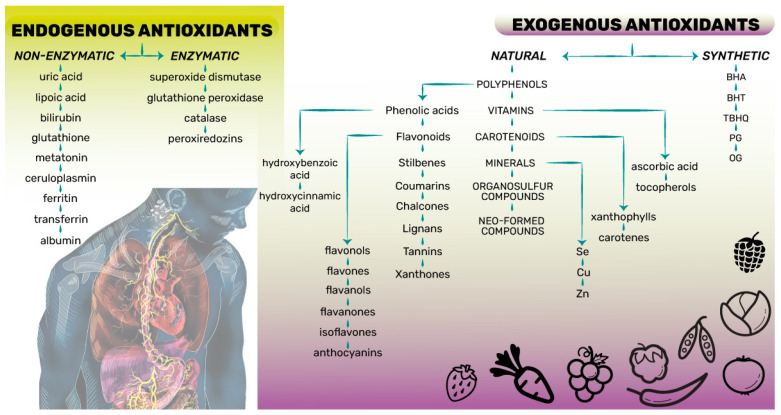
Classification of antioxidants according to source and molecule type [6,8,24,25,27,28]. BHA-Butylated hydroxyanisole, BHT-Butylated hydroxytoluene, TBHQ-Tertiary-butyl hydroxyquinone, PG-Propyl gallate, OG-Octyl gallate.

The term antioxidant refers to “any substance that delays, prevents or removes oxidative damage to a target molecule when present in low concentrations compared to that of an oxidizable substrate” [25,29]. Accordingly, an antioxidant is any molecule that can reduce or neutralize ROS and contribute to mitigating or halting oxidative stress. Endogenous antioxidants are body products consisting of enzyme systems with an antioxidant action (enzymatic antioxidants) and non-enzymatic antioxidants (Figure 1). Although endogenous antioxidants have a paramount role in the maintaining of a basic healthy antioxidant environment for cells, this antioxidant defense system is not sufficient to eliminate the free radicals that are produced; therefore, it should be supported by exogenous antioxidants supplied through food, which can be natural or synthetic (Figure 1) [8,9,24,28]. There are three lines of antioxidant defenses in the biological system [8,9,24,28]:

- The first preventive line of antioxidants suppresses or hinders free radicals’ or reactive species’ creation in cells by preventing the occurrence of reactions in which they are formed. The antioxidants involved in these reactions are predominantly endogenous enzymatic antioxidants such as superoxide dismutase, catalase, glutathione reductase, and the minerals Se, Cu, and Zn (Figure 1);
- The second repairing line of antioxidants neutralizes or scavenges free radicals or reactive species by donating an electron to them and interrupting radical chain reactions. Both endogenous non-enzymatic and natural exogenous antioxidants such as glutathione, albumin, vitamins C and E, carotenoids, and flavonoids are included in these reactions (Figure 1);
- The third line of antioxidants acts towards restoring and the reconstitution of the biomolecules and cell membranes damaged by free radicals or reactive species. These antioxidants include a complex group of enzymes (de novo enzymes) such as polymerases, glycosylases, nucleases, proteinases, proteases, and peptidases.

The corresponding antioxidant activities against ROS are positively reflected in their beneficial health effect for the prevention of oxidative stress-driven diseases. In this context, antioxidants were recognized as anti-aging, anti-cancer, anti-cataract, antidiabetic, anti-inflammatory, anti-microbial, hepatoprotective, nephroprotective, and neuroprotective compounds, and also as a defense against cardiovascular diseases [8]. However, antioxidants’ effectiveness in the early prevention of the corresponding diseases depends on various factors, including their systemic bioavailability, antioxidant concentration, suitability to be delivered to specific organ sites, and ability to perform the expected function [22]. Consequently, the disease-prevention activities of exogenous antioxidants originating from antioxidant-rich foods are proven. However, studies involving high doses of specific antioxidants have not proven beneficial effects on health [7]. This fact is called “the antioxidant paradox” and is explained by the impossibility of increasing the body’s total antioxidant capacity upon higher supplemental doses of antioxidants, reflecting an inability to change or reduce oxidative damage to key biomolecules [30]. Furthermore, high concentrations of many supplemental antioxidants can induce the overproduction of ROS, which can overwhelm the endogenous antioxidants’ defense system, indicating oxidative stress and causing toxicity [7,22]. Since an excess of both ROS and antioxidants can be detrimental, a proper balance in the oxidative-reduction system is needed for health maintenance [22].

Similarly to the human body, oxidation reactions also occur in many food products as a consequence of their exposure to air (oxygen), heat, or light during processing, storage, and distribution [27]. Food constituents such as lipids are particularly prone to oxidation, generating products with undesirable flavors or even potentially toxic substances, deteriorating the product quality. Antioxidants that are naturally present or added to food play a similar role to endogenous antioxidants in the body. By impeding lipid oxidation, they preserve the sensory attributes, texture, and safety of the food product [9,24,27]. Accordingly, antioxidants in food products were first employed as preservatives, and afterwards, abreast with their revealed role in human metabolism, antioxidants gained attention as ingredients with putative health benefits [6,7]. However, to be used as food-grade antioxidants, they need to meet the following terms [27]:

- Must be approved by regulatory bodies and have GRAS status (generally recognized as safe);
- Food product color, odor, or flavor should not be negatively affected by antioxidant addition;
- Should be effective when added at low concentrations (0.001–0.01%);
- Should be easily applicable and compatible with foods they are used in;
- Should be steady during processing and storage;
- Should be economical.

Considering increased consumer awareness regarding the link between diet and health, and the negative implications concerning synthetic antioxidant usage, the need for natural antioxidants is rising. The majority of natural antioxidants originate from fruits, vegetables, herbs and spices rich in phenolic compounds, vitamins, and carotenoids [6,9,24,27,28]. Although these antioxidant compounds are obtained from natural sources, they still present some limitations such as their imparted flavor or taste, the dosage, and possible toxicological effects that must be addressed when considering their usage as food-grade antioxidants [27].

## 3. Methods of Quantification and Identification of Antioxidant Compounds Used in Gluten-Free Bread

According to Prior et al. [31], certain requirements should be satisfied to establish a standardized method for the determination of antioxidant activity expressed by food components in the corresponding matrix, such as:

- Defined mechanism of reaction and end point in a particular food matrix with a measurable content;
- Utilization of biologically relevant free radicals to closely reflect in vivo action;
- Simple performance, and accessible chemicals and equipment;
- Reliable within samples and between-day reproducibility;
- Adaptable for measurement of both lipophilic and hydrophilic antioxidants as well as for the use of other radical sources;
- Adaptable to high-throughput analysis for routine quality control analyses.

The variety of food matrices and the structural diversity of antioxidant compounds, as well as differences in their mechanisms of action, are the main reasons for failing to meet the stated requirements. Hence, the antioxidant activity of a particular additive in the food matrix still needs to be evaluated by conducting several assays [25].

Sample preparation for corresponding analysis is also an important step which influences the accuracy of the results. The sample preparation of GFB envelopes two main steps: (a) extraction with methanol/water mixture, acidified or not [32,33], with the aid of ultrasound [32,33,34], reflux [35], simple [36,37,38] or intense stirring [39,40], or shaking [41], followed by (b) separation by centrifugation with [39] or without [34,35] subsequent fine filtration.

**Figure 2:**
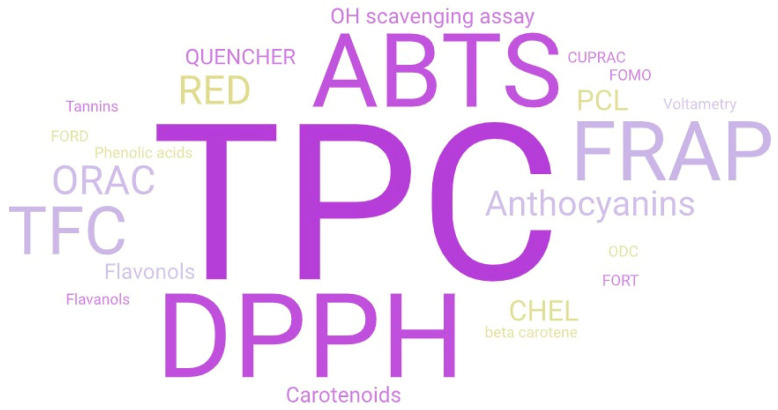
The most frequently applied methods in the determination of gluten-free bread antioxidant capacity. Generated with https://wordart.com/create (accessed on 20 September 2023). For abbreviations see Table 1 footnote.

The first authors to determine antioxidant capacity in GFB were Alvarez-Jubete et al. [39] and Wronkowska et al. [42], who did so by performing a total phenolic content (TPC) assay, 2,2-Diphenyl-1-picrylhydrazyl assay (DPPH), 2,2-Azinobis 3-ethylbenzthiazoline-6-sulfonic acid radical scavenging assay (ABTS•+), and ferric reducing antioxidant power assay (FRAP), but also by identifying polyphenolic compounds [39]. It was found that the most commonly applied methods for antioxidant quantification in GFB were, in descending order: total phenolic content (TPC) assay, 2,2-Diphenyl-1-picrylhydrazyl assay (DPPH), 2,2-Azinobis 3-ethylbenzthiazoline-6-sulfonic acid radical scavenging assay (ABTS•+), ferric reducing antioxidant power assay (FRAP), and total flavonoid content (TFC) assay (Figure 2). Other sporadically applied assays of antioxidant quantification in GFB include the oxygen radical absorbance capacity (ORAC) [37,41,43,44,45], chelating activity (CHEL) [46,47], photochemiluminescence (PCL) [48], reducing power (RED), and hydroxyl radical scavenging (•OH) [49] assays, as well as copper reducing antioxidant capacity (CUPRAC) assay [50]. The quantification of a particular class of antioxidants such as anthocyanins, flavonols [51], carotenoids [52], phenolic acids [53], or tannins [54] using spectrophotometry was also less frequently performed. In vivo antioxidant activity (FORD) and in vivo free radicals (FORT) assays of consumers’ blood were only applied by Bedrníček et al. [35]. A detailed summary regarding applied antioxidant compound quantification and identification methods in GFB, alongside the source and addition levels, is available in Table 1.

TPC or Folin–Ciocalteu reagent (FCR) represents a method based on the single electron transfer (SET) mechanism, where the yellow molybdotungstophosphoric heteropolyanion is reduced by the present phenolic compounds in alkaline conditions, resulting in a blue-colored molybdotungstophosphate that is detectable spectrophotometrically at 750–765 nm [25,31,80]. In this way, apart from TPC assay, the free and bound phenolic compound contents were evaluated [33]. Although specified conditions (proper volume ratio of alkali and FCR, optimal reaction time and temperature, monitoring of optical density at 765 nm, and gallic acid as reference standard) were proposed as guidelines for more predictable results with diminished variability [80], they were not completely adopted neither in different food products nor for determination in GFB. Namely, gallic acid is the most frequently used reference standard for TPC assay in GFB, with the expression unit being mg of gallic acid equivalent (GAE) [33,35,48,52], but ferulic acid equivalent (FAE) [42,55], catechin equivalent (CE) [57], and chlorogenic acid (CGA) [78] were also used. Furthermore, the reaction time applied for GFB enveloped a span from 20 min [34,39] or 30 min [37,65,79] to 60 min [33] or 120 min [35,52,54]. Moreover, even greater variety was observed in terms of the wavelengths at which readings were made, starting from 685 nm [60], over the 720–735 nm range [37,39], and finally at the originally proposed 750–765 nm range [35,40,41,48,79], regardless of the chosen reference standard.

DPPH is the second most frequently used method for antioxidant compound activity determination in GFB, representing a radical scavenging assay mainly based on SET mechanism [31]. The detection of the reducing ability of antioxidants toward DPPH•, which results in a colorless solution, is evaluated through spectrophotometric measurement of the absorbance reduction in a certain time frame (maximum absorbance at 515 or 517 nm) [25]. The result can be reported as the percentage of inhibition, as the inhibitory concentration (IC50, the quantity of antioxidant required to decrease the initial DPPH concentration by 50%), or, more accurately, as the equivalent concentration of the standard radical scavenging compound after absorbance interpolation in a concentration–response curve (Trolox-synthetic hydrophilic vitamin E analogue, or ascorbic acid) [25]. Regarding GFB samples, the results were most frequently expressed as a Trolox equivalent (TE) [33,35,48], i.e., Trolox equivalent antioxidant capacity (TEAC), [39,52] and, in some cases, as butylated hydroxytoluene (BHT) [32] or ascorbic acid [75] equivalents. Scarcely, results were only reported as IC50 or the percentage of inhibition [56,74]. Readings were usually performed at zero time and 30 min after the reaction at the wavelengths 515 nm [39,42,54] or 517 nm [48,50,79]. Rarely, longer or shorter reaction times [35,52] and wavelengths of 516 nm [33], 520 nm [72], or 525 nm [41] were applied.

ABTS is another radical scavenging assay which takes place according to SET but also a hydrogen atom transfer (HAT) mechanism [31]. The corresponding assay entails the generation of intensely colored ABTS•+ radical cations, by chemical or enzymatic means, and its subsequent reaction with antioxidants in the sample, which leads to an absorbance decrease, quantified spectrophotometrically at 734 nm in a fixed time frame (4–6 min) [31]. The obtained results are most commonly expressed as TEAC [31]. The stated conditions were followed by authors applying this method for GFB antioxidant capacity determination [33,48,67] (Table 1).

FRAP assay, based entirely on a SET mechanism, evaluates the reducing capacity of antioxidant compounds of a ferric ion (Fe^3+^)-ligand complex reduction to the intensely blue-colored ferrous (Fe^2+^) form under acidic conditions, quantified spectrophotometrically through the time-dependent absorbance increase (593 nm or 595 nm) [31,81]. TPTZ (2,4,6-Tripyridyl-S-triazine) has been originally applied as an iron-binding ligand; as an alternative ligand, ferrozine (ascorbic acid-reducing power evaluation) or potassium ferricyanide can be used [81]. The results are expressed as micromolar Fe^2+^ equivalents or relative to a reference antioxidant standard (Trolox or ascorbic acid). The use of FRAP coupled with other methods to measure radical scavenging ability is considered significant for the determination of the dominant reaction mechanism exhibited by antioxidant compounds [31]. Concerning GFB, results were dominantly reported as TE [35,39,48,50,60,72], but also as Fe^2+^ equivalents [37,41,45,55] and ascorbic acid equivalents [71,75]. Variations regarding applied wavelengths were also noticed; however, the majority of the readings were performed at 593 nm [34,35,50,63,72,75] over 595 nm [37,60]. Even more pronounced differences among GFB samples were observed concerning the reaction time starting from 4 or 5 min [33,34] over 30 min [35,37] and even over 40 min [39].

TFC was an often-used method for antioxidant compound determination in GFB samples according to the aluminum chloride colorimetric assay based on the formation of Al^3+^-flavonoids complex, detectable spectrophotometrically in the wavelength range 410–440 nm depending on the used reference standard (catechin, rutin, or quercetin) [82]. Additionally, the introduction of other chemicals in the assay, such as sodium nitrite, shifts the detection wavelength to 510 nm [82]. The results regarding GFB are quantified from a calibration curve based on a reference standard such as catechin equivalents (CE or CATE) [33,72], rutin equivalents (RE) [37,66], or quercetin equivalents (QE) [38,63], but also as GAE [74]. Considering the readout wavelength, 415 nm was commonly selected [37,38] even in cases where sodium nitrite was added [33]. Similarly, 510 nm-wavelength readings were selected for determining both the presence [74] and absence [72] of sodium nitrite. The reaction time was another experimental condition which differed following the determination in GFB samples from immediate reading [72,74] and reading after 30 min [33,37,38]. The corresponding method clearly shows a high degree of diversity when applied in GFB samples compared to other aforementioned methods, and raises doubts about the reliability of the results.

Most recently, the so-called QUENCHER (QUick, Easy, New, CHEap, Reproducible) approach, as a direct and simple assay for antioxidant capacity measurement [83], was applied in GFB samples [41,45,78]. Considering that the GFB, like most food products, is a matrix which contains free as well as bound hydrophilic and lipophilic antioxidant compounds, their comprehensive extraction using a single solvent or solvent mixture and single extraction method is hardly achievable. Therefore, the resulting extracts fail to reflect the actual total antioxidant capacity of the food product concerning its physiological resembling as well as changes emerging from the product’s storage [6]. The QUENCHER approach relies on the antioxidant capacity determination, which excludes the extraction and hydrolysis of samples but is, however, adaptable to conventional methods (ABTS, DPPH, FRAP, CUPRAC, ORAC) and represents a step forward in standardization of the variety of methods presented across studies [6]. The basic steps of the QUENCHER procedure are described in Gökmen et al. [83]. Hitherto, the QUENCHER approach in GFB samples was applied on DPPH [41,45] and ABTS [41,45,78] assays (Q-DPPH and Q-ABTS, respectively) following the same experimental parameters and reference standards but on a small amount of finely ground solid sample (~10 mg).

The profiling of antioxidant compounds in GFB samples was not so often analyzed together with already mentioned methods for antioxidant capacity determination. Only several studies reported results on both antioxidant capacity and individual antioxidant compounds’ identification and quantification employing high-performance liquid chromatography (HPLC) coupled with different detectors [33,36,39,66,72] (Table 1). Among the determined individual antioxidant compounds in GFB were, usually, phenolic acids (gallic, ellagic, syringic, *p*-coumaric, ferulic, chlorogenic, and caffeic acid) [33,36,66,72] and flavonoids (quercetin and quercetin derivatives, kaempferol derivatives, rutin and catechin, luteolin, and apigenin) [36,39,66,72] or solely rutin and quercetin [35,43,46]. In addition, tocopherols and carotenoids [33] were rather scarcely determined.

Previously stated observations hamper the possibility of meaningful comparison of the obtained results regarding antioxidant compounds’ activity in GFB with various plant-based additives and impose a necessity of corresponding methods’ standardization. However, the antioxidant compound activity methods’ path to standardization is still vague and requires the acquisition of adequate conditions that are particular to each method and sample. Therefore, noteworthy details to consider when selecting the most adequate antioxidant compound detection method are the presence of particular antioxidant compounds in the food matrix based on the antioxidant source and the antioxidant reaction mechanism unfolding in the food matrix. This should pave the way toward the utilization of a comprehensive group of methods for antioxidant activity determination in food as well as in the GFB matrix.

## 4. Basic Sources of Antioxidant Compounds in the Gluten-Free Bread Production

**Figure 3:**
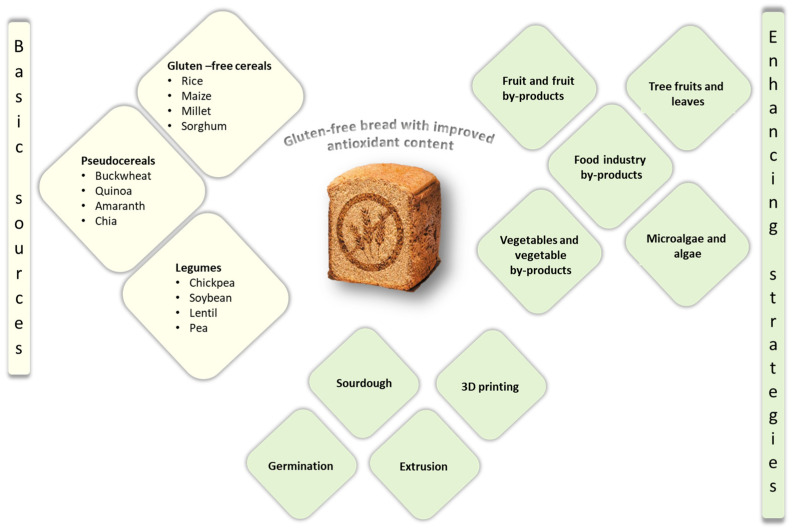
Basic sources and enhancing strategies applied to obtain antioxidant-enriched gluten-free bread.

Although GFB is still considered nutritionally inferior compared to its gluten-containing counterparts, some improvements have been achieved when the usage of starches and refined flours was replaced with the use of alternative and whole-grain GF flours. Commonly used nutrient-rich flours include those from GF cereals, pseudocereals, and legumes [3] (Figure 3). Accordingly, these flours also became basic sources of antioxidant compounds in GFB production (Table 2). Antioxidant compounds are concentrated mainly in the outer layers of the mentioned GF grains, affecting the grain color, which is related to the antioxidant compounds’ type and content. Thus, the grain color reflects the overall antioxidant activity of grains [5]. Subclasses of polyphenols such as phenolic acids, flavonoids (predominantly anthocyanins), and tannins are the most frequently present in GF cereal, pseudocereal, and legume flours, alongside carotenoids (β-carotene) and vitamins which exert antioxidant activity (tocopherols, tocotrienols). In addition to the aforementioned GF flours, and in line with the sustainable development goals, food industry by-products (pomace, peel, leaves, husk) (Table 1) are new rising sources of antioxidant compounds for direct application in GFB production [34,35,48,51,66,69,70,71,78] and will be discussed in more detail in Section 5.2.

Antioxidant compounds are frequently accompanying compounds of other plant components such as dietary fibers and proteins [37,84,85]. Usually, antioxidant compounds in GFB production are not used in an isolated (extracted) purified form, which may be beneficial in exerting higher health-promoting effects than when being applied alone [86]. However, it should also be stated that, paradoxically, some antioxidant compounds (polyphenols, tannins) could, in some respect, be considered antinutrients because of their interference with protein digestion [32]. Likewise, results reported on antioxidant compounds’ content and antioxidant activity in GF flours are difficult to compare considering the various extraction techniques, solvents, and conditions applied, as well as the several standards utilized for the expression of the results, as discussed previously (Section 3) [5]. Additionally, the existence of antioxidant compounds (especially phenolic acids) in GF grains mainly in a bound form also brings difficulties in their accurate quantification [87].

### 4.1. Gluten-Free Cereals as Basic Sources of Antioxidant Compounds in Gluten-Free Bread Production

#### 4.1.1. Rice

Rice flour represents the most frequently applied cereal flour in GFB production thanks to characteristics such as a bland taste, white color, good digestibility, and hypoallergenic properties [88]. Apart from the most commonly used white rice, rice grains have different kernel colors including brown, orange, violet, and black which exert a better nutritional composition in terms of dietary fiber, amino acids, minerals, and antioxidant compounds [89,90]. Paramount antioxidant compounds in rice are polyphenols, whose type and concentration, to a great extent, influence the kernel color and antioxidant activity [89,91,92]. Correspondingly, black and purple rice is linked to anthocyanins, whilst a red rice color is linked to tannin (proanthocyanidins) content [92,93]. Pigmented rice flours, particularly violet rice, expressed a high TPC and anthocyanins content, and accordingly, high antioxidant activity determined by FRAP and ORAC methods (Table 2), which is in line with a previously established correlation between rice antioxidant activity and its kernel color [92], as well as its anthocyanins content [89]. Anthocyanins commonly present in pigmented rice flours are cyanidin 3-*O*-glucoside, malvidin 3-*O*-arabinoside, petunidin 3-*O*-arabinoside, and pelargonidin 3-*O*-(6″-malonyl-glucoside) [89], which can slow down the starch digestion process and decrease the risk of type-2 diabetes and obesity by acting as competitive inhibitors of amylolytic enzymes [89,94].

#### 4.1.2. Maize

The second basic ingredient that is most frequently used in GFB production is maize flour and its starch [95]. White maize could be more preferred for use in GFB production compared to yellow, with its characteristic yellow color and typical flavor [2], as it is more appreciated in countries with traditional bakery products that use yellow maize flour [96]. Additionally, the most convenient yellow maize flours for GFB production, according to de la Hera et al. [97], are those with a coarser particle size. Although both white and yellow maize kernels contain carotenoids, flavonoids, and anthocyanins to some extent, remarkably higher contents of the aforementioned antioxidant compounds are found in orange, red, purple, blue, and black maize varieties (Table 2) [98,99,100]. A high quantity of anthocyanins is found in dark-red, purple, blue, and black maize, whilst orange and red-yellow varieties were found to be high in carotenoids (lutein and β-carotene) [98,99,100]. A relationship between the colorizing antioxidant compound content (anthocyanins, flavonoids, and carotenoids) and antioxidant activity was established, implying higher antioxidant activity for dark-colored maize varieties (Table 2) [98,99,101]. However, differences in the antioxidant activities of red, purple, and blue maize kernels mainly derive from the specific compositions of anthocyanin derivatives, considering the presence of 13 anthocyanins in colored maize, with cyanidin 3-glucoside (Cy-3-Glu) being the most dominant form [98]. Regarding the phenolic acids, ferulic, *p*-coumaric, and *o*-coumaric acids were found in colored maize varieties [98]. Despite the great antioxidant potential of dark-colored maize varieties, their usage as an ingredient in GFB production is scant [58].

#### 4.1.3. Millet

The unique composition of millet and, consequently, derived milled flour, in terms of biologically active components such as antioxidant compounds (tannins, flavonoids, phenolic acids, and β-carotene), vitamins (B1, B2, B3, and E), and minerals (K, Ca, P, Mg, Fe, and Zn) followed by a high content of dietary fiber (13.1%) [71], implies the potential of millet flour inclusion in GFB production [32,55,60,71]. In addition, other types of phytochemicals such as γ-amino butyric acid were detected in millet varieties (foxtail and barnyard), specifically in their germinated grains [87] (pp. 85–103). The most abundant phenolic compounds in millets, likewise in all cereal grains, are phenolic acids and flavonoids [87] (pp. 85–103). Besides the aforementioned antioxidant compounds, condensed tannins (proanthocyanidins/procyanidins) were additionally found in the brown-colored finger millet varieties [102]. Phenolic acids found in diverse millet varieties are present in different quantities and include both hydroxybenzoic acids (protocatechuric and vanillic acid) and hydroxycinnamic acids (ferulic, caffeic, coumaric, and sinapic acid), as well as their derivatives, existing predominantly in the bound form [103]. Generally, finger millet is considered the most abundant in flavonoids with identified catechin, gallocatechin, epicatechin, epigallocatechin, taxifolin, vitexin tricin, luteolin, myricetin, quercitin, kaemferol, narigenin, and daidzein, where the majority of them are present in the free form [103]. The TPC and antioxidant activity of millets are regarded as intermediate compared to sorghum, and are higher compared to major cereals (wheat and rye) [104] (Table 2). Due to its high amount of dietary fiber and phenolic compounds, millet flour inclusion in GFB formulation could spawn numerous health benefits involving a reduction in the occurrence of lipid disorders, cardiovascular diseases, hyperglycemia, and delays in gastric emptiness [60].

#### 4.1.4. Sorghum

Sorghum is another GF cereal that is insufficiently used even though it possesses a nutrient composition that is comparable to other cereals [55]. The superiority of sorghum over major cereals and edible plants is reflected in a vast spectrum of varied bioactive constituents, including phenolic compounds as paramount antioxidants, with some of them uniquely found in sorghum only (3-deoxyanthocyanin) [105]. The dominant phenolic compounds in sorghum are phenolic acids, flavonoids (anthocyanins, flavones, and flavanones), and tannins (proanthocyanidins), whilst additionally present are carotenoids (lutein, zeaxanthin, and β-carotene), phytosterols, policosanols, and trace amounts of α-tocopherol and α-tocotrienol, which also contribute to sorghum’s antioxidant activity (Table 2) [54,59,105,106] (pp. 21–54). The contents of tannins and 3-deoxyanthocyanin pigments concentrated in sorghum grain’s pericarp determine the kernel color (ranging from brown to red and black) and antioxidant activity [105,107].

**Table 2:** Total phenolic and flavonoid content (TPC and TFC), and antioxidant activity of basic gluten-free flours.

Raw Material	Variety	Total Phenolic Content [mg GAE/g d.b.]	Total Flavonoid Content [See Units in Footnote]	DPPH [See Units in Footnote]	FRAP [See Units in Footnote]	ABTS [See Units in Footnote]	ORAC [See Units in Footnote]	Anthocyanins [See Units in Footnote]	Reference
Gluten-free cereals									
Rice		0.104	/	0.66 ± 8.16 ^i^	/	8.20 ± 138.39 ^i^	/	/	[41]
		1.39 ± 0.13	/	3.52 ± 1.02 ^j^	/		/	/	[56]
		0.063–0.069	0.01–0.06 ^c^	/	/	0.013–0.017 ^w^	/	/	[108]
	white	0.20	/	/	/	/	/	/	[90]
	brown	0.72	/	/	/	/	/	/	[90]
	red	1.11 ± 0.22	/	/	/	0.012 ± 0.24 ^x^	/	/	[93]
	ermes	0.68 ± 1.6	/	/	2.00 ± 7.5 ^s^	/	14.44 ± 8.9 ^z^	/	[89]
	nerone	3.41 ± 9.9	/	/	12.85 ± 29.4 ^s^	/	72.28 ± 68.1 ^z^	1.22 ± 1.3 **	[89]
	orange	0.76 ± 0.7	/	/	2.57 ± 26.4 ^s^	/	25.51 ± 79.6 ^z^	0.006 ± 0.2 **	[89]
	wild	0.82 ± 1.4	/	/	1.96 ± 11.3 ^s^	/	31.82 ± 35.7 ^z^	0.014 ± 0.1 **	[89]
	violet	5.00 ± 23.7	/	/	20.90 ± 47.1 ^s^	/	117.84 ± 63.2 ^z^	2.48 ± 5.9 **	[89]
	black	2.40 ± 3.6	/	/	9.51 ± 29.4 ^s^	/	39.59 ± 90.7 ^z^	0.66 ± 1.3 **	[89]
Maize		0.295	/	4.89 ± 153.80 ^i^	/	13.41 ± 210.15 ^i^	/	/	[41]
	white	6.75 ± 0.67 ^a^	/	34.41 ± 3.34 ^j^	18.05 ± 1.16 ^t^	/	/	0.45 ± 0.16 ^t^	[99]
	white	5.23 ± 0.33	0.25 ± 0.004 ^d^	/	/	/	/	n.d.	[98]
	lemon yellow	5.78 ± 0.037	0.28 ± 0.02 ^d^	/	/	/	/	n.d.	[98]
	yellow	5.4 ± 0.01	0.281 ± 0.002 ^d^	/	/	/	/	n.d.	[98]
	yellow	2.01	0.75 ^e^	3.81 ^k^	5.18 ^k^	3.98 ^k^	/	/	[63]
	yellow	11.25 ± 0.94 ^a^	/	46.73 ± 4.55 ^j^	32.11 ± 1.85 ^t^	/	/	0.75 ± 0.21 ^t^	[99]
	orange	5.81 ± 0.14	0.29 ± 0.002 ^d^	/	/	/	/	n.d.	[98]
	red-yellow	6.01 ± 0.23	0.268 ± 0.006 ^d^	/	/	/	/	0.0025 ± 0.06 **	[98]
	red	6.04 ± 0.20	0.267 ± 0.003 ^d^	/	/	/	/	0.015 ± 0.002 **	[98]
	red	16.45 ± 1.76 ^a^	/	67.57 ± 1.94 ^j^	49.02 ± 1.66 ^t^	/	/	9.35 ± 0.93 ^t^	[99]
	dark red	6.11 ± 0.16	0.27 ± 0.003 ^d^	/	/	/	/	0.696 ± 0.003 **	[98]
	purple	34.25 ± 1.26 ^a^	/	78.32 ± 2.27 ^j^	54.96 ± 1.46 ^t^	/	/	12.45 ± 1.07 ^t^	[99]
	light blue	10.53 ± 0.06	0.34 ± 0.013 ^d^	/	/	/	/	0.378 ± 0.005 **	[98]
	blue	10.39–13.31	/	/	1.52–2.03 ^n^	/	/	0.65–1.05 **	[101]
	dark blue	7.35 ± 0.5	0.31 ± 0.017 ^d^	/	/	/	/	0.60 ± 0.007 **	[98]
	multicolored	4.49 ± 0.29	0.20 ± 0.013 ^d^	/	/	/	/	0.14 ± 0.002 **	[98]
	black	/	/	/	10.96 ^u^	/	/	5.375 ^u^	[100]
Millet	pearl	/	/	0.73 ± 0.00 ^l^	/	/	/	/	[32]
		1.39 ± 13.3	/	23.83 ± 0.67 ^m^	/	21.4 ± 0.43 ^y^	/	/	[104]
Sorghum		4.13 ± 9.3	/	195.8± 8.82 ^m^	/	51.7 ± 0.57 ^y^	/	/	[104]
	white	0.52 ± 0.2	/	/	1.46 ± 7.5 ^s^	/	22.36 ± 63.1 ^z^	/	[89]
	red	1.08 ± 5.1	/	/	3.24 ± 9.9 ^s^	/	47.35 ± 59.5 ^z^	/	[89]
Pseudocereals									
Buckwheat		3.23 ± 14.1	/	6.20 ± 28.1 ^n^	4.36 ± 12.8 ^n^	/	/	/	[39]
		4.98 ± 0.11	/	53.08 ± 0.82 ^j^	/	/	/	/	[56]
		7.25 ± 0.2	153 ± 12 ^f^	8.80 ± 0.52 ^o^	2.15 ± 3.5 ^n^	/	/	/	[109]
	light	3.32 ± 4.76	/	1.36 ± 0.01 ^p^	/	/	/	/	[46]
	wholegrain	4.15 ± 13.8	/	1.26 ± 0.09 ^p^	/	/	/	/	[46]
	Common	29.3 ± 0.5	1.0 ± 0.2 ^g^	/	9.1 ± 1.6 ^v^	/	139.3 ± 33.4 *	/	[37]
	Tartary	72.8 ± 0.5	22.2 ± 0.3 ^g^	/	40.5 ± 4.4 ^v^	/	450.3 ± 57.7 *	/	[37]
Quinoa		0.717 ± 5.5	/	0.577 ± 1.7 ^n^	0.921 ± 1.7 ^n^	/	/	/	[39]
		2.8 ± 0.1	92 ± 9 ^f^	6.22 ± 0.2 ^o^	0.59 ± 1.5 ^n^	/	/	/	[109]
		5.22 ± 0.17	/	60.14 ± 0.76 ^j^	/	/	/	/	[56]
		2.26	0.43 ^e^	5.67 ^k^	5.49 ^k^	2.02 ^k^	/	/	[63]
	white Spanish quinoa	/	/	4.56 ± 0.03 ^q^	3.65 ± 0.30 ^q^	4.57 ± 0.28 ^q^	/	/	[110]
	white Bolivian Real quinoa	/	/	3.43 ± 0.14 ^q^	3.36 ± 0.11 ^q^	4.01 ± 0.25 ^q^	/	/	[110]
	white Peruvian quinoa	/	/	1.94 ± 0.11 ^q^	2.37 ± 0.28 ^q^	3.88 ± 0.19 ^q^	/	/	[110]
	red Bolivian Real quinoa	/	/	5.01 ± 0.04 ^q^	4.57 ± 0.17 ^q^	7.76 ± 0.17 ^q^	/	/	[110]
	black Bolivian Real quinoa	/	/	4.77 ± 0.02 ^q^	4.22 ± 0.00 ^q^	5.72 ± 0.34 ^q^	/	/	[110]
Amaranth		21.2 ± 2.3		28.4 ± 1.3 ^n^	55.3 ± 1.6 ^n^				[39]
		2.55 ± 0.20		18.46 ± 0.93 ^j^					[56]
		2.71 ± 0.1	65 ± 8 ^f^	3.60 ± 0.34 ^o^	0.39 ± 1.2 ^n^				[109]
Chia		16.4 ± 0.9	1.1 ± 0.2 ^g^	/	11.0 ± 1.4 ^v^	/	131.0 ± 13.3 *	/	[37]
Legumes									
Chickpea		0.24–0.42	0.17–0.40 ^c^	/	/	0.03–0.04 ^w^	/	/	[108]
		1.22–1.67 ^b^	0.021–0.1 ^h^	/	/	/	/	0.04–0.066 **	[111]
		0.93–10.84	/	/	0.73–1.13 ^v^	/	8.74–52.2 ^z^	/	[112]
Soybean		0.95–1.39	0.33–0.57 ^c^	/	/	0.07–0.08 ^w^	/	/	[108]
		0.98–2.62	/	/	1.24–1.96 ^v^	/	22.2–86.8 ^z^	/	[112]
	yellow	13.35–14.64	0.39–0.50 ^g^	3.91–11.74 ^n^	6.43–10.86 ^n^	5.93–12.28 ^n^	/	n.d.	[113]
	black	16.46–21.49	0.74–0.90 ^g^	26.60–28.36 ^n^	35.87–55.02 ^n^	20.21–23.05 ^n^	/	0.58–1.03 **	[113]
Lentil		2.22	/	21.00 ± 23.96 ^i^	/	188.45 ± 45.22 ^i^	/	/	[40]
	red	4.68 ± 0.3	/	64.26 ± 2.84 ^r^	/	/	/	/	[114]
Pea		0.07–0.22	0.33–0.48 ^c^	/	/	0.01–0.02 ^w^	/	/	[108]

^a^ FAE mg/g DW; ^b^ mg GAE/g extract; ^c^ mg rutin/g fw; ^d^ mg CE/g dm; ^e^ mg QE/g dw; ^f^ µg CE/g dw; ^g^ mg RE/g dw; ^h^ mg RE/g extract; ^i^ Q- Direct antioxidant properties (μmol TE/g dm); ^j^ Inhibition %; ^k^ Mmol TEAC/g; ^l^ mmol BHT/kg dm; ^m^ µmol BHT/g 10 min; ^n^ mgTE/g dwb; ^o^ mmol TE/kg dw; ^p^ IC50 mg dmb/mL; ^q^ mg TE/g fw; ^r^ EC50, mg/mL; ^s^ μmol GAE/g; ^t^ mg/g dw; ^u^ µmol/g fw; ^v^ mmol Fe2+E/g; ^w^ nmol Trolox/g fw; ^x^ mmol Trolox/g; ^y^ µmol TE/g 3 min; ^z^ μmol TE/g; * mmol GAE/g; ** mg CGE/g; n.d.—not detected.

Correspondingly, pigmented sorghum varieties containing tannins express higher antioxidant activity and TPC compared to white varieties (Table 2) [89,106,115] (pp. 21–54). Sorghum possesses a higher share of flavonoids compared to other cereal grains in which major phenolic compounds are primarily phenolic acids. The phenolic acids present in sorghum include ferulic acid, the main acid that is present, and gallic and vanillic acids, which are extractable in a relatively high proportion [105,106] (pp. 21–54). The majority of the antioxidant compounds present in sorghum can be preserved upon thermal processing and thus imparted in sorghum-derived products, expressing health benefits that are distinctive from other cereals and which are linked to its bioactive properties that are relevant in cancer and cardiovascular disease prevention and reduced chronic inflammation and oxidative stress [106] (pp. 21–54). In this regard, sorghum tannins were the most studied, revealing dual behavior as natural ingredients to reduce the caloric influence of starch and also as antinutrients due to the formation of poorly digestible complexes with proteins [106] (pp. 21–54).

### 4.2. Pseudocereals as Basic Sources of Antioxidant Compounds in Gluten-Free Bread Production

#### 4.2.1. Buckwheat

Buckwheat could be considered as the most commonly used pseudocereal in GFB production [37,39,42,61], characterized by high-quality proteins with balanced essential amino acids (rich in lysine, leucine, and arginine), as a good source of dietary fiber and lipids rich in unsaturated fats [37,39,42,116] (pp. 161–177). Important micronutrients that are additionally present in buckwheat are minerals and vitamins (B1, B2, and vitamin E), alongside a significant amount of health-promoting bioactive compounds such as saponins, phytosterols, squalene, fagopyritols, and polyphenols [39,42]. Apart from polyphenols, other antioxidant compounds present in buckwheat are carotenoids, glutathione, and melatonin (non-enzymatic endogenous antioxidants) [42]. Buckwheat represents an exceptional source of polyphenols with strong antioxidant activity and is one of the most-studied pseudocereals regarding phenolic composition [39,89]. The polyphenols present in buckwheat include phenolic acids and flavonoids such as rutin, quercetin, catechin, epicatechin, epicatechin 3-gallate, isoquercetin, orientin, vitexin, isovitexin, isoorientin, and kaempferol-3rutinoside [32,46,90,116] (pp. 161–177). The corresponding flavonoids (predominantly rutin) are responsible for buckwheat’s remarkably greater TPC and antioxidant activity as determined by DPPH compared to other cereals and legumes (Table 2) [32,37,39,90]. The content and composition of buckwheat’s phenolic compounds vary depending on the species and growing conditions [116] (pp. 161–177). Consequently, Tartary buckwheat has a higher flavonoid content, TPC, and antioxidant activity than common buckwheat (Table 2), which is mainly attributable to its greater rutin content; however, it is rarely consumed due to its bitter taste originating from enzymatic degradation of the highly presented rutin [37,117]. Buckwheat is the only pseudocereal that contains rutin in quantities greater than most fruits, vegetables, and grain crops, representing a significant source of this flavonoid [116,118] (pp. 161–177). The importance of buckwheat rutin is reflected in the strong antioxidant ability of its metabolic product quercetin to scavenge free radicals and chelate metals, thus impeding lipid peroxidation [119]. Additionally, in bread made with Tartary buckwheat flour, a decline in rutin was established, while the quercetin content increased and retained stability during processing [120], revealing promising health outcomes upon buckwheat usage in GFB production.

#### 4.2.2. Quinoa

The usage of quinoa in GFB is recommendable considering its well-balanced nutritional composition and high amounts of unsaturated fatty acids, vitamins (E, B, and C), minerals (Ca, Fe, Mg, Mn, Cu, and K), dietary fiber, and polyphenols [55,110,121] (pp. 37–60). Particularly, quinoa possesses high-quality proteins with amino acid compositions (including lysine, tryptophan, and cysteine) that are close to ideal according to FAO recommendations and deficient in cereals [121,122]. Except for polyphenols, quinoa represents a good source of betalains (betaxanthins), exerting high-antioxidant and free-radical-scavenging properties [123]. Phenolic acids (caffeic, ferulic, *p*-coumaric, *p*-hydroxy-benzoic, vanillic, galic, syringic, *o*-cumaric, chlorogenic, and rosmarinic acid) and flavonoids (kaempferol, quercetin, rutin, isoquercetin, neohesperidin, and hesperidin) are the most prevalent polyphenols in quinoa [63,110,124] (pp. 105–129). Diverse quinoa varieties (white, red, and black) express different TPC and antioxidant activities (Table 2), demonstrating the dependence of the amount of polyphenolic compounds on the genotype (variety and cultivar), soil, environmental conditions, plant maturity, harvest, and post-harvest conditions [110]. As in other grains, higher TPC, TFC, and antioxidant activities were associated with dark-colored varieties (Table 2) [110,124] (pp. 105–129). Additionally, a good positive correlation was found among the TPC, TFC, and antioxidant activities as assayed by DPPH, ABTS, and FRAP, implying that quinoa’s antioxidant activity is derived from the present phenols and flavonoids [110].

#### 4.2.3. Amaranth

Similarly to quinoa, a well-balanced nutritional composition is also a characteristic of amaranth, strongly related to its protein content and quality, which are higher than in other cereal grains (high in lysine, methionine, cysteine, and histidine) [125] and accompanied by, mainly, polyunsaturated fatty acids, with linoleic, oleic, palmitic, and stearic acid being the most abundant [126] (pp. 137–159). Abreast with the aforementioned major nutrients, minerals, vitamins, dietary fibers, and various antioxidant compounds were found in amaranth in considerable amounts [126] (pp. 137–159). The antioxidant compounds found in amaranth include phenolic acids, flavonoids, tannins, tocopherols, and betalains, detected only in pink amaranth varieties [127]. Among phenolic acids, protocatechuic, vanillic, 4-hydroxybenzoic, *p*-coumaric, syringic, caffeic, sinapic, cinnamic, ferulic, salicylic, and gallic acid were identified with concentrations that varied depending on the genotype, species, and location. [128,129]. Rutin is the main flavonoid compound detected in amaranth, followed by nicotiflorin and vitexin, and isovitexin present in lower quantities [121,130]. Amaranth’s TPC and TFC are comparable to quinoa’s (Table 2), but despite this fact and greater quantities of phenolic compounds with putative high antioxidant potential (such as rutin), its antioxidant activity is rather low compared to quinoa (Table 2), with no significant correlation between amaranth’s TPC and antioxidant activity [109,131].

#### 4.2.4. Chia

Chia seeds have gained popularity in recent years as a new nutrient-rich ingredient authorized by The European Commission [132] (pp. 34) owing to their remarkable quantity of ω-3 α linolenic acid and ω-6 linoleic acid, their protein content that is higher than in commonly used grains, and the fact that they contain all of the essential amino acids, especially leucine, lysine, valine, and isoleucine [133]. In addition, chia seeds exhibit a high dietary fiber content and the ability to expel a natural exudate in an aqueous solution in the form of branched polysaccharide, alongside a substantial quantity of antioxidant compounds (Table 2) [37]. Phenolic compounds are the most abundant antioxidants in chia seeds, are present in a free or bound form, and include phenolic acids (rosmarinic, protocatechuic, caffeic, chlorogenic, ferulic, and gallic acids), flavonols (myricetin, quercetin, and kaempferol), and isoflavones (daidzin, glycitin, genistin, glycitein, and genistein) [134]. Similar TPCs were found in different chia seeds from Mexico regardless of the cultivation place (Jalisco or Sinaloa) [135]. Although chia seeds’ TFC and antioxidant activity as determined by FRAP and ORAC assays were comparable to common buckwheat but lower than in Tartary buckwheat (Table 2), they are characterized as a new source of isoflavones in the human diet [37].

### 4.3. Legumes as Basic Sources of Antioxidant Compounds in Gluten-Free Bread Production

#### 4.3.1. Chickpea

Chickpea is a well-recognized protein, lipid, dietary fiber, and mineral source containing phenolic compounds, soyasaponins, and volatile aliphatic hydrocarbons as phytochemicals [136,137]. However, anti-nutritional factors such as hemagglutinins, trypsin inhibitors, phytic acid, and tannins are also chickpea constituents that may counteract chickpeas’ utilization in GFB production [138]. The phenolic compounds occurring in chickpeas include phenolic acids (23 hydroxybenzoic acids and 13 hydroxycinnamic acids) and flavonoids (29 flavonols, 12 isoflavones, and anthocyanins) [111,137]. Two distinct types of chickpeas (kabuli and desi) exert differences in the seeds’ coat color, which is darker for desi chickpeas [139]. Similarly to GF cereals and pseudocereals, chickpea seeds’ coats’ darker pigmentation is linked to higher TPC, TFC, and anthocyanin contents, indicating stronger antioxidant activity (Table 2) [111]. Correspondingly, when investigating different chickpea seed fractions (coat, embryonic, and cotyledon), significantly high antioxidant activity in terms of DPPH was observed in the seed’s coat fraction, which was primarily ascribed to flavonols (quercetin, kaempferol, and myrcetin) and anthocyanins (cyanidin, petunidin, and delphinidin). Phenolic acids such as ferulic, protocatechuic, caffeic, chlorogenic, and *p*-coumaric acids were major carriers of antioxidant activity in the cotyledon fraction [140]. Additionally, the isoflavones genistein and daidzein, known as phytoestrogens involved in the reduction of risk factors for cardiovascular disease and cancer occurrence, were present in higher quantities in chickpea seeds’ embryonic fraction compared to cotyledon and seed coat fractions [140].

#### 4.3.2. Soybean

Soybean seeds are well known as an extraordinary source of protein and, additionally, contain considerable amounts of lipids that are rich in unsaturated fatty acids and carbohydrates, with calcium and iron being main minerals that are present [141]. Bioactive constituents found in soybean seeds are oligosaccharides, phytosterols, saponins, phytic acid, and phenolic compounds such as phenolic acids, anthocyanins, and isoflavones [113,142]. Anthocyanins were determined only in black soybean varieties, contributing to the seeds’ coloration as well as their antioxidant activity (Table 2) [113]. It is considered that isoflavones comprise about 72% of all phenolic compounds in soybean seeds [143], with daidzin and genistin being the most abundant [113,142], whilst eight phenolic acids including chlorogenic, *p*-hydroxybenzoic, caffeic, protocatechuic, ferulic, *p*-coumaric, gallic, and cinnamic acid were also detected in soybeans [113]. Soybean seeds and their derived products are considered the major source of isoflavones in the human diet, gaining more pertinence in recent years after the increasing evidence of soybean isoflavones’ positive health effects [142]. Greater TPC, TFC, isoflavone, and anthocyanin contents were reported for black soybean seeds when compared to yellow soybean seeds, and consequently, black seeds expressed higher antioxidant activities in terms of DPPH, ABTS, and FRAP (Table 2). Accordingly, a significant positive correlation was established between soybean seeds’ antioxidant activity and TPC, TFC, isoflavone, and anthocyanin contents [113].

As regards the other legumes, lentils and peas were to some extent applied in GFB production (Figure 3), and their antioxidative potential is presented in Table 2 [41,108,114]. As for other food products, the presence of antinutrients is the main obstacle to the greater incorporation of legumes in GFB production.

Many factors can influence the antioxidant content of raw materials used in GFB production, and additionally, on the way from flour to bread, thermal and other processing conditions can greatly modify antioxidant compound content and activity, which will be discussed in more detail in the following section.

## 5. Strategies to Improve the Content of Antioxidant Compounds in Gluten-Free Bread

Celiac patients who have not yet started a GF diet are exposed to significantly higher oxidative stress due to the disruption of the pro-oxidant–antioxidant balance in the small intestinal mucosa by gliadin ingestion, causing ROS overproduction [11,12]. Conducted studies reveal an altered antioxidant capacity in celiac patients that is ascribed to glutathione consumption and a decrease in glutathione peroxidase and glutathione reductase activity, as well as the activity of other enzymes. Additionally, greater oxidative stress is linked to more progressive mucosal damage in celiac patients; thus, a GF diet rich in natural antioxidants could be an effective way to accomplish mucosal healing and prevent the further development of non-communicable diseases and cancer [10,13,14,15]. However, GFB, as a paramount element in the GF diet, consists of refined flour, starch, and hydrocolloids and requires further enrichment with natural antioxidant-rich ingredients. An increase in the antioxidant supply for celiac patients on a GF diet can be accomplished by the introduction of new plant-based raw materials and additives with strong antioxidant properties in GFB formulations (Table 3) such as the already mentioned GF cereal, pseudocereal, legume flours [5], agricultural and food industry by-products, residues and extracts thereof [34,35,48,51,65,66], and algae and microalgae [75,76,77] (Figure 3). Apart from plant-based additives, promising strategies for improving the antioxidant compound content in GFB can be GF grain pre-treatment involving germination, the application of sourdough technology, extrusion and 3D printing, or an approach combining plant-based additives with previously mentioned technologies [39,40,41,52,55,60,63,68] (Figure 3). Studies investigating the antioxidant compound content and activity in GFB formulations are outlined in Table 3, together with the plant source, applied quantity, and technology.

Difficulties in determining the real antioxidant content and activity of produced GFB are encountered, likewise for GF flours and additives; these are attributed to various extraction techniques, solvents, conditions, and standards used for the expression of the results, as well as the existence of antioxidant compounds in a predominantly bound form [5]. Furthermore, it must be considered that many factors can alter phenolic content and antioxidant activity on the way from raw material to GFB, including thermal processing (baking), the activity of enzymes, and fermenting microorganisms [89]. Concomitantly, the resulting changes in antioxidant compounds’ stability and structure ultimately affect their biological activity and health outcomes, which are still scarcely investigated to date.

Thus, it is hard to compare the antioxidant contents and activities among various GFBs and differentiate the influence of plant-based additives concerning their origin, quantity, and variations in the formulation’s composition. Still, in the following sections, an overview of the corresponding additive effects and other strategies for overcoming antioxidant compound deficiency in GFB will be given.

### 5.1. Effect of Bread-Making on Antioxidant Compounds Content in Gluten-Free Bread

The influence of bread-making, which includes mixing, fermentation, and baking, on antioxidant compounds’ content and activity in the resulting GFB is ambiguous. Conducted studies disclosed both increments [33,45,57,58,66] and decreases [37,39,46,53] in the TPC, TFC, and antioxidant activity of GFB. Pertinent mechanisms involved in this process are still insufficiently explored; however, some conclusions can be drawn. The baking step has the principal impact (both positive and negative) on antioxidant compounds’ content, whilst lesser effects of mixing and yeast fermentation were observed [58,66,144]. Estimated losses in phenolic compounds during baking are not greater than 60% [39,145], and their chemical structure is the main factor that determines their stability [144]. Flavonols are considered more resistant to thermal degradation compared to phenolic acids, while anthocyanins are the most labile [53,146]. Besides the chemical structure, other factors affecting phenolic compounds’ stability are the type of substrate, processing temperature, and duration in the case of flavonoids, as well as the pH and presence of enzymes when referring to anthocyanins [39,53]. Accordingly, losses of TPC, TFC, and antioxidant activity in GFB are a corollary of polyphenols’ thermal, enzymatic, and oxidative degradation upon baking [39,147], and the additional formation of complexes with polysaccharides and proteins [49,66]. On the other hand, an increase in the antioxidant compounds’ content and activity after baking is commonly ascribed to:

- Weakening of the cell wall matrix enabling a release of phenolic acids from bounded forms [45,58];
- The synthesis of Maillard reaction products, which are substances exerting antioxidant properties [37,39,45,46,55,57];
- The presence of other antioxidant compounds beyond polyphenols such as vitamins with antioxidant activity not detectable by the applied methods [57];
- The decomposition or conversion of complex molecules (tannins, quercetin derivatives, rutin) to simpler phenolic compounds (mostly phenolic acids and quercetin derivatives) [33,58,66].

Regarding the Maillard reaction products, baking triggers the formation of furosine (FUR) and N-ε-(carboxymethyl)lysine (CML), which are considered harmful, but also melanoidins with antioxidant, anti-inflammatory, and prebiotic activities [58,148]. The melanoidins’ antioxidant activity mainly occurs by an SET mechanism and contributes to the final activity as determined by radical scavenging methods and chelating methods (FRAP, DPPH, ABTS, •OH) [149]. Additionally, plant-based antioxidant compounds were recently regarded as a possible solution in combating the health risks arising from the formation of already mentioned advanced Maillard reaction products (FUR, CML) [58].

In a complex system such as GFB formulation, the aforementioned reactions takes place simultaneously, causing both increases and decreases in antioxidant compounds’ content and activity in the resulting GFB. The reactions that prevail further determine the GFB antioxidant compounds’ content and activity compared to the starting flour. In some cases, the greater the starting flour antioxidant content, the greater the losses observed in GFB were; however, the obtained GFB still had a higher antioxidant activity compared to control GFB [39,52].

### 5.2. Plant-Based Additives as Improvers of Antioxidant Compounds Content in Gluten-Free Bread

Poor GFB nutritional quality and the avoidance of food that is naturally high in antioxidants and without gluten are putative barriers to a higher antioxidant intake in celiac patients. Great variations are present in the TPC, TFC, and anthocyanins contents and antioxidant activity of GFB and are hardly comparable between the investigated samples considering the used extraction techniques, solvents, quantification methods, and standards, as already mentioned [5]. Likewise, there is a great diversity in GFB constituents [55]. However, the TPC and antioxidant activity of GFB prepared from commercial mixtures, according to the literature, is 0.98 mg GAE/g and 87.24 mg CGA/g as determined by direct ABTS•+ assay (QUENCHER), and 10.9% as determined by DPPH, respectively [78,79]. Additionally, the TPC, DPPH, and FRAP values reported for GFB (8.8 mg GAE/100 g, 5.6 mg TE/100 g, 47.6 mg TE/100 g, respectively) are lower than those reported for wheat bread (29.1 mg GAE/100 g, 14.1 mg TE/100 g, 81.7 mg TE/100 g, respectively) [39], justifying the need for GFB enrichment. Accordingly, the most applicable and feasible strategy for GFB enrichment with antioxidant compounds is the inclusion of plant-based raw materials and additives that are naturally high in antioxidants (Table 3).

Many studies have investigated the influences of plant-based antioxidants and received identical outcomes, reflected in enhanced GFB antioxidant contents and activity. However, the extent of the GFB antioxidant enhancement was significantly dependent on the plant-based additives’ origin, their richness in antioxidant compounds, and the amount added (Table 2 and Table 3) [33,35,39,48,57,66,70,77]. Correspondingly, GF cereals, pseudocereals, and legumes were used as single raw materials in GFBs [39,55], as additives in quantities from 3–50% [42,45,46,50,56,57], and in the form of mixtures combining different flours [33,37]. Among GF cereals, pseudocereals, and legumes, buckwheat (especially Tartary buckwheat) stood out as the most preferable GFB constituent in terms of antioxidant content and activity, predominantly ascribed to its high rutin amount (Table 2 and Table 3) [37,39,42,46,57]. As regards the incorporation level of other plant-based antioxidants, with increasing addition levels of fruit and fruit by-products 1–15% [65,66,67,69,70], vegetable and vegetable by-products 5–10% [35,51,53], and herbs and tree fruits and leaves 1–35% [33,38,40,49], a greater antioxidant enrichments of GFB were achieved (Table 3). Recently, algae and microalgae as well as agricultural and food industry by-products and residues (broccoli leaf, coffee husk, and silverskin) have arisen as sources of antioxidant compounds intended for GFB production to contribute to the accomplishment of sustainable development goals. The quantity of corresponding “sustainable” additives applied in GFB ranged from 2 to 10% for powders [48,75,76,77,78,79], and 25 to 100% when used as liquid extracts for water replacement [34]. Even when applied in lower amounts compared to GF cereals, pseudocereals and legumes, fruits, vegetables, and algae, their by-products and residues are evidenced to be promising antioxidant sources in GFB production (Table 3). Greater TPC and TFC contents in enriched GFB entailed greater antioxidant activity, as confirmed by high correlation coefficients established between both the total and individual antioxidant compounds and their activity (Table 3) [51,66]. Despite the great improvement achieved in antioxidant content, the sensorial perception of enriched GFB should not be compromised, and the bioavailability of GFB antioxidant compounds is another constraint that should be addressed as discussed in Section 7 and Section 8.

**Table 3:** Total phenolic and flavonoid content (TPC and TFC) and antioxidant activity of gluten-free breads after the introduction of enhancing strategies including different plant-based additives, application of pre-treatment, and processing technologies.

Antioxidant Source	Addition Level (%)	Pre-Treatment/Technology	TPC [See Units in Footnote]	DPPH [See Units in Footnote]	ABTS [See Units in Footnote]	FRAP [See Units in Footnote]	TFC [See Units in Footnote]	Reference
Gluten-free cereals, pseudocereals and legumes								
Wholegrain rice flour	100		0.70 ± 0.10 ^a^	10.50 ± 0.11 ^j^	6.97 ± 0.10 ^l^	0.98 ± 0.05 ^v^		[55]
Maize flour	10		0.16 ± 0.02 ^b^	/	3.4 ± 0.2 ^s^	/	0.067 ± 0.006 ^b^	[57]
Brown millet flour	100		1.8 ± 0.10 ^a^	19.24 ± 0.10 ^j^	15.07 ± 0.10 ^l^	2.06 ± 0.07 ^v^	/	[55]
Wholegrain millet flour and Wholegrain millet extruded flour	50	Extrusion	108.26 ± 0.001 ^c^	76.9 ± 1.02 ^j^	/	215.2 ± 1.9 ^w^	/	[60]
Wholegrain sorghum flour	100		3.87 ± 0.11 ^a^	35.01 ± 0.10 ^j^	54.51 ± 0.12 ^l^	3.67 ± 0.09 ^v^	/	[55]
White sorghum flour	85		0.412 ± 0.021 ^d^	/	0.135 ± 0.018 ^o^	0.003 ± 0.02 ^p^	/	[36]
Amaranth	50		0.138 ± 0.0 ^e^	0.103 ± 0.002 ^k^	/	0.61 ± 0.062 ^k^	/	[39]
Amaranth flour	10		0.31 ± 0.03 ^b^	/	5.26 ± 0.15 ^s^	/	0.105 ± 0.007 ^b^	[57]
Amaranth, buckwheat and quinoa flour	15, 30, 45	Sourdough	1.70–2.06 ^d^	6.81–14.59 ^j^	/	/	/	[56]
Buckwheat flour	10		0.64 ± 0.05 ^b^	/	3.4 ± 0.2 ^s^	/	0.192 ± 0.02 ^b^	[57]
Buckwheat	50		0.65 ± 0.031 ^e^	0.59 ± 0.039 ^k^	/	1.48 ± 0.046 ^k^	/	[39]
Sprouted buckwheat	100	Germination	1.16 ± 0.018 ^e^	0.77 ± 0.025 ^k^	/	2.64 ± 0.036 ^k^	/	[39]
Wholegrain buckwheat flour	30, 45		25.74–30.08 ^d^	/	/	/	/	[43]
Dehulled buckwheat flour	10, 20, 30, 40		0.42–1.22 ^a^	0.76–2.56 ^l^	1.70–4.12 ^l^	/	/	[42]
Buckwheat hulls	3, 6		0.006–0.18 ^e^	0.40–0.60 ^k^	1.91–2.61 ^k^	0.135–15.1 ^x^	/	[45]
Quinoa flour	100		3.98 ± 0.15 ^a^	32.85 ± 0.11 ^j^	19.32 ± 0.12 ^l^	2.53 ± 0.09 ^v^	/	[55]
Quinoa	50		0.307 ± 0.003 ^e^	0.168 ± 0.7 ^k^	/	0.714 ± 0.028 ^k^	/	[39]
Extruded lentil flour	15	Extrusion	0.303 ± 0.013 ^e^	2.28 ± 28.80 ^l^	7.53 ± 1.18 ^l^	0.069 ± 0.009 ^x^	/	[41]
Germinated sweet lupin and fenugreek mixtures	5, 10, 15, 20	Germination	/	5.71–6.40 ^m^	2.45–3.17 ^m^	5.65–6.45 ^m^	/	[63]
Fruit and fruit by-products								
Acerola fruit powder	1, 2, 3, 4, 5		4.4–10.1 ^e^	67.8–231.9 ^n^	34.1–89.4 ^n^	/	/	[65]
Apple pomace	5, 10, 15		0.036–0.22 ^e^	/	1.97–3.21 ^k^	/	0.08–0.22 ^y^	[66]
Defatted blackcurrant seeds	5, 10, 15		0.10–0.12 ^f^	/	1.34–2.01 ^o^	/	/	[67]
Defatted strawberry seeds	5, 10, 15		0.30–0.71 ^f^	/	3.16–5.60 ^o^	/	/	[67]
Extruded sour cherry pomace and rice flour	10	Extrusion	59.4–308.7 ^g^	/	1.817–2.297 ^t^	/	11.7–97.3 ^z^	[68]
Grape seed flour	3, 6, 9		3.63–5.92 ^d^	/	/	5.75–9.75 ^q^	/	[69]
Pomegranate seed powder	2.5, 5, 7.5, 10		1.29–2.47 ^e^	11.97–29.39 ^n^	5.16–6.22 ^n^	/	/	[70]
Rosehip powder, rosehip encapsulate	7	3D printing	0.46–0.81 ^d^	0.71–113.5 ^o^	/	/	/	[52]
Vegetables and vegetable by-products								
Freeze-dried red and purple potatoes	5		0.173–0.351 ^h^	1.995–2.113 ^k^	2.865–3.590 ^k^	/	0.080–0.147 ^y^	[53]
Red potatoes pulp	5, 7.5, 10			/		/		[51]
Purple potatoes pulp	5, 7.5, 10		0.14–0.39 ^h^	/	9.5–39.4 ^k^	/	0.019–0.057 *	[51]
Broccoli leaf powder	5		1.25 ^e^	0.95 ^l^	1.77 ^l^	/	/	[48]
Fried red onion	5		1.5 ± 0.06 ^e^	0.85 ± 0.02 ^k^	/	0.96 ± 0.02 ^k^	/	[35]
Dried red onion	5		1.87 ± 0.01 ^e^	1.23 ± 0.03 ^k^	/	1.43 ± 0.01 ^k^	/	[35]
Red onion peel	5		5.28 ± 0.11 ^e^	4.70 ± 0.02 ^k^	/	6.36 ± 0.02 ^k^	/	[35]
Okara	30		1.34 ± 0.024 ^d^	0.49 ± 8.58 ^o^	0.87 ± 0.025 ^k^	1.19 ± 0.043 ^q^	/	[71]
Herbs								
Hemp inflorescence	1, 2, 3, 4, 5		0.30–0.65 ^e^	1.66–3.23 ^l^	/	1.60–3.00 ^l^	0.06–0.16 *	[38]
Tree fruits and leaves								
Acorn flour	23, 35	Sourdough	4.541–6.810 ^e^	0.055–0.076 ^p^	0.072–0.143 ^p^	0.046–0.069 ^p^	/	[40]
Acorn flour	23, 35		0.613–0.848 ^d^	0.037–0.043 ^p^	0.066–0.073 ^p^	0.041–0.064 ^p^	5.39–6.30 **	[72]
Carob fiber (commercial)	1, 2, 3, 4, 5		7.5–9.1 ^e^	/	42.5–66.8 ^n^	/	/	[47]
*Moringa oleifera* leaves powder	2.5, 5, 7.5, 10		2.03–2.39 ^e^	10.60–31.62 ^n^	4.72–7.51 ^n^	/	/	[49]
Microalgae and algae								
Microalgae *Tetraselmis chuii*	4		0.24 ^d^	3.22 ^q^	/	0.47 ^q^	/	[75]
Microalgae biomass	4		4.38–5.47 ^d^	2.59–3.16 ^r^	/	/	/	[76]
Ethanol-treated microalgae biomass	4		1.93–3.92 ^d^	0.80–2.09 ^r^	/	/	/	[76]
Brown algae powder	2, 4, 6, 8, 10		3.7–4.7 ^e^	/	157.6–248.7 ^n^	216.5–375.9 ^n^	/	[77]
Food industry by-products								
Coffee silverskin extract	2.5		254.92 ± 7.73 ^i^	/	288.27 ± 3.57 ^u^	/	/	[78]
Coffee husk extract	2.5		121.12 ± 6.12 ^i^	/	129.39 ± 1.80 ^u^	/	/	[78]
Ground green coffee parchment	2		1.07 ± 1.16 ^d^	65.6 ± 1.6 ^j^	/	/	/	[79]
Flaxseed oil cake extract	25, 50, 75, 100		0.203–0.234 ^e^	0.852–0.945 ^l^	0.890–1.128 ^l^	0.568–0.731 ^l^	/	[34]

^a^ mg FAE/g dw; ^b^ g CE/kg dm; ^c^ µmol GAE/g; ^d^ mg GAE/g; ^e^ mg GAE/g dw; ^f^ mg/g; ^g^ mg CE/kg; ^h^ mg CE/g dw; ^i^ mg CGA/g; ^j^ Inhibition %; ^k^ mg TE/g dw; ^l^ µmol TE/g dm; ^m^ mmol TEAC/g; ^n^ EC50 mg/mL; ^o^ mg TE/g; ^p^ mmol TE/g dw; ^q^ mg AAE/g; ^r^ mg vit C eq/g dry extract; ^s^ mmol TE/kg dm; ^t^ μM TE/kg; ^u^ mg CGA/g; ^v^ μmol Fe^2+^/g dw; ^w^ μmol Fe^2+^/g; ^x^ mmol Fe^2+^/g dw; ^y^ mg Rutin/g dm; ^z^ mg Rutin/kg; * mg QE/g dm; ** mg CE/g.

### 5.3. Germination as a Pre-Treatment to Improve the Antioxidant Compounds Content in Gluten-Free Bread

As a natural and traditional bioprocessing method, germination is frequently applied for enhancing and tailoring the nutritional value of cereals, pseudocereals, and legume seeds for diverse uses in food production. The usage of such a simple, inexpensive, and environmental-friendly bioprocessing method triggers the activation of enzymes (proteases, amylases), enabling the enhanced digestibility and bioaccessibility of nutrients and bioactive compounds as well as increased antioxidant activity following antinutrient content decreases [39,150]. In addition, mostly favorable changes in seeds’ structures and techno-functional properties, as well as flavor, are introduced, reflecting positively on the final product’s technological quality.

Regarding antioxidant-enriched GFB, germinated brown rice [44] and white sorghum [54] were included in the formulations as well as the pseudocereals buckwheat, amaranth, and quinoa [39]. Legumes were less-applied in the germinated form, and only lupine and fenugreek were used for GFB enrichment [63]. The conditions for germination differed among seeds and included ambient temperatures (25, 27, and 28 °C) for the mentioned cereals and legumes [44,54,63], while 10 and 18 °C were applied to buckwheat and amaranth/quinoa, respectively [39]. Cereals were germinated for 48 h [44,54], while for legumes and pseudocerals, the time was prolonged to 72 h and 96 h, respectively [39,63]. When germinated, buckwheat, amaranth, and quinoa exhibited at least doubled TPC compared to their seeds, alongside increased values for FRAP, while the DPPH remained in the same range [39]. Consequently, the GFB produced from germinated buckwheat had higher TPC, DPPH, and FRAP values compared to control GFB and wheat counterparts (Table 3) [39]. Furthermore, the TPC and TFC in GFB containing brown rice and white sorghum germinated for 48 h were found to increase and were associated with bound phenolic release by enzymes’ participation in the degradation of cell wall polysaccharides and proteins [44,54]. Although not ambiguous for every seed, a positive relation between the increase in the germination time and the TPC was established [44]. In general, germination as a pre-treatment exhibits affinity toward TPC increases; still, both the seed variety and germination time are conditions which contribute in a great extent to the corresponding changes.

### 5.4. Technologies Applied to Improve the Antioxidant Compounds Content in Gluten-Free Bread

#### 5.4.1. Sourdough

Sourdough fermentation, as an ancient biotechnological process, was first explored regarding the flavor, rheology, technological quality, and shelf life of cereal-based breads. Nowadays, the focus has shifted towards nutritional advantages offered by sourdough fermentation such as reduced glycemic index, enhanced fiber solubility, minerals bioavailability, and protein digestibility, likewise the release of bound phenolic compounds and increased antioxidant capacity [64,151]. Sourdough can be obtained by spontaneous fermentation, but more often were explored defined starter cultures combined with baker’s yeast as more relevant for industrial bread-making due to minimized variation in the microbial composition and controllable fermentation resulting in a product of a more standardized quality [64,151].

Regarding antioxidant-enriched GFB, sourdough as a potential enhancement strategy was used on white sorghum flour [54], pseudocereals flour-buckwheat, quinoa and amaranth [56], cereals–pseudocereals mixtures [152,153], yellow pea flour [64], and acorn flour [40]. Without further application in GFB, sourdoughs from refined rice flour, wholegrain quinoa, and buckwheat flour were produced [151].

For sourdough preparation, different sources of microorganisms were used, starting from spontaneous fermentation and bakers’ yeast [56], including self-cultivated lactic acid bacteria (LAB) strains, namely *Lactobacillus paracasei* ssp. *Paracasei*, *Lactobacillus parabuchner*, *Lactobacillus brevis*, and *Leuconostoc mesenteroides* ssp. *mesenteroides* [152], as well as *Pediococcus pentosaceus* SA8, *Weissella confuse*, and *Pediococcus pentosaceus* LD7 [54] likewise commercial sourdough (organic rye liquid sourdough) [40] and commercial bacterial cultures such as *Lactobacillus reuteri* DSM 20016, *Lactobacillus fermentum* DSM 20052, or *Lactobacillus brevis* DSM 20054 (DSMZ, Braunschweig, Germany) [64]. Furthermore, lyophilized water and milk kefir grains with inulin solution (20% *w*/*v*) as a cryoprotective agent were also applied in GFB [153]. The sourdough was included in the antioxidant-enriched GFB formulation as a liquid [40] or lyophilized [153], usually replacing 20% of the water and/or batter base [40,54,56,64]. The preparation of sourdough envelopes [54,56,153]:

- Propagation of the selected LAB culture in DeMan, Rogosa, and Sharpe (MRS) broth and yeast on Yeast Extract Peptone Dextrose (YEPD) agar under defined conditions;
- Sterilization of the fermentation medium, i.e., flour blend and water (105 °C, 10 min);
- Inoculation with starter culture and fermentation, commonly for 24 h at 25 or 30 °C according to the culture’s affinity.

In general, the TPC content of antioxidant-enriched GFB was found to increase with sourdough inclusion (Table 3) [54,56,64]. GFB antioxidant activity followed the same tendency [56,64,153] or stayed unchanged (Table 3) [40]. The corresponding increase was attributed to the action of LAB enzymes reflecting bound phenolic compounds’ release and their conversion to associated derivatives, as well as an improved solubility coming from acidification contributing to facilitated extractability [40,64]. The applied starter culture and treated flour or blends were identified as the main factors affecting the extent of the induced effect on phenolic compounds’ content [40,64,153]. Additionally, the emphasized remark was the complementary impact of sourdough fermentation and included the antioxidant compounds’ source [40,64].

Among the tested starters, *P. pentosaceus* SA8 [54], *L. brevis* [64], and water kefir grains [153] were selected as the most convenient for GFB in terms of TPC, DPPH, individual phenolic acids, flavonoids, and tannins enhancement. In GFB enriched with rice-millet-based sourdough obtained by *L. brevis* alone and accompanied with yellow pea flour, increased values of 4-hydroxybenzoic acid and vanillic acid were detected [64]. Furthermore, liquid- and solid-state fermentation conducted with water kefir grains on a mixture of chickpea, quinoa, and buckwheat flours resulted in increased gallic acid, caffeic acid, epicatechin, and isorhamnetin quantities. Nevertheless, the same fermentation substrate with added okara, subjected to solid-state fermentation by water kefir grains, resulted in a reduction in myricetin, quercetin 3-β-D-glucoside, and apigenin content. This suggests that the amount and composition of individual bioactive compounds, besides the starter culture and substrate composition, depends also on the conducted fermentation type [153].

#### 5.4.2. Extrusion

Extrusion technology represents a tool for various flours’ transformation using joint effects originating from applied high temperature and mechanical shear at a relatively low flour moisture content [41]. Changes provided by extrusion are multiple and include [41,60,68]:

- Enzyme inactivation and microbial population reduction;
- Antinutrients content reduction;
- Increase in dietary fiber solubility;
- Reduction or increase in bioactive polyphenols content due to degradation or release from dietary fiber;
- Gelatinization and degradation of starch and protein aggregation.

The corresponding changes lead to enhancement in techno-functional and nutritional properties of the flour constituents, namely increased water adsorption capacity, digestibility of starch and proteins, and bioactive compounds’ bioavailability [41,60].

Extruded rice, millet, and maize flours were recently applied in GFB formulations [60] in combination with various shares of sour cherry pomace [68] and lentil [41] to improve its antioxidant properties (Table 3). A single screw lab-scale extruder (Brabender mod.KE19 20 DN, Duisburg, Germany) [41,68] and twin screw extruder (Evolum HT25, Clextral Inc., Firminy, France) [60] operating at screw speeds of 150–190 rpm and 500 rpm, respectively were employed. The usually used circular die diameter was 3.8–4 mm, while the length/diameter ratio varied depending on the extruder type. The examined temperatures were 80 and 120 °C for the rice-sour cherry pomace blend [68], 110, 120, and 130 °C for the maize-lentil blend [41], and 140 °C for the wholegrain millet [60], with moisture contents ranging from 14 to 20%. The obtained extrudates were subsequently room- or oven-dried (25 or 45 °C) and ground before inclusion in the GFB formulation [41,60].

The general conclusion derived from the conducted studies regarding GFB implies that a rise in extrusion temperature (120, 130, or 140 °C) results in an increase in TPC, TFC, total phenolic acids, and anthocyanins content, likewise antioxidant activity enhancement (DPPH, FRAP, ABTS) regardless of the included flour origin (Table 3) [41,60,68]. Even greater enhancement was noted by the direct measurement (QUENCHER) of Q-DPPH and Q-ABTS [41]. Such an outcome was associated with several changes such as enhanced phenolic compounds extraction due to cell structure modification induced by high extrusion temperature, the hydrolysis/release of bound polyphenols accompaniers of fiber and/or protein moieties, and Maillard reaction products’ formation and their accession in reaction with FCR [41,68]. Furthermore, the stability of the phenolic compounds originating from wholegrain millet subjected to extrusion (140 °C, high shear) was established [60], while those present in sour cherry pomace and considered thermally labile were further stabilized by extrusion with starch from rice flour as a protective agent [68]. In summary, extrusion as an enhancement strategy certainly deserves attention concerning antioxidant compounds’ integrity and functionality protection.

#### 5.4.3. 3D Printing

3D printing technology represents an emerging technology that supports the development of foods with customized designs and textures, including tailored nutritional contents. One of the most interesting applications for this technology is in developing appealing food personalized for population groups with special nutritional needs and conditions, namely vegans, as well as celiac, non-celiac wheat sensitivity (NCWS), irritable bowel syndrome (IBS), dysphagia, and other patients [154,155]. The most prominent advantage when applying 3D printing is the relatively low heat treatment during the process; hence, it represents a promising tool to fabricate food enriched with bioactive compounds from fruits and vegetables or their by-products without compound degradation [156] (pp. 117–149).

As an extrusion-based process, 3D printing encompasses the layer-by-layer deposition of viscoelastic edible ink (consisting of water, protein, and supporting additional ingredients) with the aid of a printer nozzle [157]. The lack of gluten and reduced protein amount in GF cereals are the main obstacles in formulating GF edible ink capable of sustaining the dimensions and shape of the printed form. Hence, careful selection of the GF edible ink ingredients, familiarity with their rheological properties, and the optimization of printing conditions (nozzle speed and flow rate) are the main challenges to combat when creating GFB or other GF products with 3D printing as an enhancement strategy. Although food product 3D printing has been extensively studied, 3D printing in terms of GF products based on flour is still underexplored [157], especially regarding antioxidant-enriched GFB.

An incipient study concerning 3D printed GFB enriched with rosehip powder and encapsulate (7%) was performed by Matas et al. [52]. The authors printed GFB in a rectangular base that was 7 cm long and 3 cm wide but with a variable height (1, 2, and 3 cm) using a 20 mm/s nozzle speed, 1.7 mm layer height, and 60% rectilinear infill (100% in the first layer). Although an increment in TPC, DPPH, and total carotenoids content was observed due to rosehip presence, the 3D printing effect on the corresponding results was not fully elucidated (Table 3). Nevertheless, it was emphasized that knowing the input edible ink rheological parameters is crucial for successful 3D printing concerning the stability and definition of the GFB printing lines. The resulting conclusion was that the rheological properties of the studied formulation exhibit a deformational effect on GFB printed at a 3 cm height [52]. Nevertheless, the evolution of 3D printing as an enhancing strategy regarding GFB still needs to be comprehensively explored.

## 6. Role of Antioxidant Compounds in Gluten-Free Bread’s Technological Quality

The crucial trigger for the purchase and consumption of many food products as well as GFB relies on an appealing visual impression. Considering technological quality attributes such as pale crust and crumb color, reduced volume, undeveloped crumb structure, and crumbly texture, it is evident that GFB lags compared to wheat counterparts as a consequence of the used ingredients [158]. Alongside the enhanced nutritional profile, plant-based antioxidants’ inclusion in GFB induces changes in the appearance, color, texture, aroma, and taste of such GFB products, which can negatively affect consumer acceptability [5]. Hence, it is essential to determine the maximal tolerable addition values of plant-based antioxidants depending on their origin. Commonly assessed indicators of antioxidant compound-enriched GFB’s technological quality are crust and crumb color, volume and specific volume, crust and crumb texture parameters, primarily hardness, and, to a smaller extent, bake loss, crumb structure, and water activity, as minutely discussed in the following sections. Additionally, changes in the crust and crumb color and hardness were monitored during storage (up to 3 or 7 days) [43,45,48,73]. Nevertheless, results regarding the GFB shelf-life related to oxidative stability [79] are scarce, as well as 5-hydroxymethylfurfural (HMF) content [79], which falls within the food safety segment of the developed product. The estimation of the acrylamide content, which represents a rising concern due to its harmful effect on humans, was only covered in one study [51], although its formation is enabled in this type of foodstuff. Estimation of the antioxidant-enriched GFB’s technological quality was usually performed 1 h [45,55], 2 h [65,75], or 24 h after baking [34,50].

### 6.1. Effect of Antioxidant Compounds Source and Addition on Crust and Crumb Color of Gluten-Free Bread

An appealing color perception is of the utmost importance in product-purchase decision-making. The addition of antioxidant compounds is recognized as an effective path toward the enhancement of the conventional pale crust and crumb color of GFB originating from its refined ingredients. Depending on the antioxidant compound’s origin, besides the natural pigments’ presence which is commonly related to crumb color, GFB formulations with these compounds included can contribute to the promotion of Maillard and caramelization reactions occurring during baking [65,72], primarily reflecting on changes in the crust color. The crust and crumb color were usually reported through parameters of the CIE Lab color space, including L* (lightness), a* (redness-greenness), and b* (yellowness-blueness), and sporadically accompanied by parameters such as total color difference (∆E*), whiteness index (WI) of the crumb, and the browning index (BI) of the crust obtained by calculations [34,49,50]. Other color parameters of enriched GFB such as the hue (h) and chroma of the color (C*) were reported only in the most recent studies [38,52]. Nevertheless, L* is regarded as the foremost parameter for GFB’s crust and crumb color description [159].

The color of GFB was evaluated by different colorimeter types (CR-400, CM-600d, or CM-3500d Chroma Meter, Konica Minolta; 4Wave CR30-16, Planeta; Chromameter HP-2132) [38,75,77] and a spectrophotometer (ColorFlex, HunterLab; ColorEye XTH) [34,35,50]. The color measurements were performed in several replicates, namely 6, 9, or 15 on crust [48,61,79] and 3, 6, 9, or 15 on crumbs [38,48,75,79] by placing the instrument in the middle point on the top of the loaf crust and the middle point of the central bread slice (thickness 2 cm) [48]. The exact conditions during measurement such as the illuminant and observation angle were sporadically listed [61,75,79].

Regardless of the antioxidant compounds’ origins (cereals and pseudocereals, fruit and fruit by-products, vegetables and vegetable by-products, herbs, tree fruits and leaves, microalgae and algae, or residues) (Figure 3), their incorporation in GFB resulted in a decrease in its lightness (L*) in both crust and crumb [35,38,48,50,57,61,70,75]. The corresponding decrease represents a consequence of naturally occurring pigments such as anthocyanins, tannins, chlorophylls, carotenoids, and other phenolic compounds in the added plant-based antioxidants; this is a simple strategy to attain the desired appealing product color. The addition of wholegrain red sorghum flour contributed to lower crumb L* values, indicating a darker appearance as confirmed by the reported BI increase. GFB made from 100% light and wholegrain buckwheat flour induced a reduction in the crumb L* parameter for approximately 8 and 39%, respectively, while, when coupled with chia flour, reduction reached from 19 to 40% compared to the control flour mixture [61]. Similarly, for GFB from whole common buckwheat and whole Tartary buckwheat, a 33–36% decrease in crumb lightness was reported compared to the wheat counterpart [37]. Furthermore, the lightness of the crust was also reduced in the corresponding GFB, except in the case of light buckwheat flour, where a slight increase was noted [37,61]. Regarding fruit and vegetables as antioxidant compound sources, acerola, rosehip, extruded sour cherry pomace, onion, and onion peel additions resulted primarily in crumb darkening [35,52,65,68], while the crust evinced a decreasing as well as an increasing trend [52,65]. The same tendency for crumb lightness was observed upon microalgae biomass [75,76] and brown algae [77] inclusion, as well as the inclusion of Hemp inflorescence [38], acorn flour [72], and residues such as broccoli leaf powder [48] and green coffee parchment [79]. It should be emphasized that, in addition to the L*, significant changes were noted in the parameters a* and b* when adding fruit, microalgae, and residues such as broccoli leaves into GFB due to their original coloration [48,52,76]. Moreover, the corresponding changes are further amplified by increases in the incorporated antioxidant compound amounts [65,75].

### 6.2. Effect of Antioxidant Compounds Source and Addition on the Specific Volume of Gluten-Free Bread

As another paramount visual characteristic that is crucial for customers purchasing the final product, the GFB loaf volume represents a result of the combined action of several factors, such as the content of amylose, the surface active compound presence (polar lipids and proteins), the dietary fiber presence, and the batter rheological properties [50,55,160,161].

A widespread method for the determination of loaf volume, therefore, also antioxidant-enriched GFB loaf volume, is rapeseed displacement, as listed by the approved AACC method 10-05.01 [37,75,79]. Millet seed was also used instead of rapeseed [48,50,60]. Nevertheless, fewer studies also applied specific devices for GFB volume evaluation, namely a Volscan Profiler (Stable Micro Systems, Godalming, UK) [45,57,68] and Volumetric Analyzer (Perten Instruments) [32]. Additionally, the GFB specific volume obtained as a ratio of the bread volume (cm^3^) to the bread weight (g) was reported in the majority of studies, enabling actual comparison of the baking performance results across studies [33,43,61] (Table 4). The determined antioxidant-enriched GFB specific volumes ranged from 0.6 to 4.78 cm^3^/g for breads based on various cereals [32,36,55] and from 1.34 to 3.63 cm^3^/g for pseudocereal inclusion [37,43,45] (Table 4). Regarding fruit and fruit by-product additions, the display of the results as volume is more common [65,67] (Table 4). The addition of flours from tree leaves (*Moringa oleifera*) and fruits (acorn, chestnut) yielded GFB-specific volumes in the range of 1.85–3.27 cm^3^/g [33,49,73] while, with microalgae incorporation, the obtained values were in the range of 1.95–2.96 cm^3^/g [76]. Fewer studies reported a specific volume of GFB supplemented with residues, and the range was 2.39–3.65 cm^3^/g [34,48,79] (Table 4).

Compared to wheat counterparts, a specific volume-depressing effect was observed when GFB was made from pregelatinized rice, millet, and buckwheat [32] or supplemented with 10% chia flour [37] or 30% acorn and chickpea flour mixtures with a higher share of acorn [33]. Furthermore, the addition of 10% amaranth, maize, or chestnut flour [57,73], 10% *Moringa oleifera* leaves powder [49], and 4% microalgae biomass and ethanol-treated microalgae biomass [76] yielded antioxidant-enriched GFB with a reduced specific volume compared to control GFB. Nevertheless, primarily with fruit and fruit by-product inclusions, namely acerola fruit powder, pomegranate seed powder, and extruded sour cherry pomace [65,68,70], the volume or specific volume of the enriched GFB was raised, and the same trend was noted with the addition of Hemp inflorescence [38], acorn flour [72], carob fiber [47], brown algae powder [77], and by-products such as broccoli leaf powder [48] and flaxseed oil cake extract [34]. Moreover, an 85% addition of white sorghum flour resulted in GFB with the largest specific volume among the added antioxidant compounds summarized in Table 4 [36]. Overall, besides the antioxidant compounds’ source, the enriched GFB volume and consequently the specific volume were also affected by the extent of the antioxidant compounds’ addition level [50,65,67], as well as that of other used ingredients in the formulation [36], which can be associated with observed discrepancies in the reported results. Another very important factor to highlight is the purity of the antioxidant compounds included so far in GFB formulations. Extract or isolate of the particular antioxidant compound was rarely included conversely for antioxidant compounds with other accompanying compounds that were predominantly applied. The accompanying components are most often dietary fibers, which can evince both positive and negative effects on the GFB volume depending on their type, as described in more detail by Djordjević et al. [161]. In conclusion, the increase or decrease in the volume of antioxidant-enriched GFB is not solely related to the antioxidant compound itself, but represents a joint effect of the antioxidant compound and accompanying compounds such as dietary fibers, the level of its inclusion, and other ingredients used in the formulation.

### 6.3. Effect of Antioxidant Compounds Source and Addition on the Crumb and Crust Texture of Gluten-Free Bread

The widely accepted method for evaluation of GFB textural properties is the texture profile analysis (TPA), which can be conducted on several texture analyzer devices with associated software (TA.XTplus or TA.HDplus Texture Analyser, Stable Micro Systems Products Ltd., Godalming, UK; ZWICK Z020/TN2S, ZwickRoell, Ulm, Germany; INSTRON 3342 universal texture analyzer, Norwood, MA, USA; TVT 6700 Texture Analyzer, Perten Instruments, Waltham, MA, USA; MLFTA apparatus, Guss, Strand, South Africa) [32,36,48,50,55,70]. The TPA test envelopes the double compression of a bread slice or pieces of a bread slice in a reciprocating motion which emulates the action of the jaw and delivers a two-bite texture profile curve [48] corresponding to the crumbs. From the obtained curves, parameters reflecting the textural properties of GFB such as hardness (firmness), springiness, cohesiveness, chewiness, and resilience are further calculated [38,48,50,52,61,79]. In addition, a puncture test corresponding to crust hardness was also sporadically performed [52,73,79]. The used texture analyzers for the crumb texture assessment of antioxidant-enriched GFB were equipped with 5, 25, 30, and 50 kg load cells [43,50,52,75] while, in a significant portion of studies, this information was neglected. The accessories involved were aluminum or acrylic cylinder probes [75,79] in diameters of 10 mm [60,75], over 20 mm [57,67,68], 30 mm [70,77], and from 35 mm [48,73,79] up to 75 mm [61], which performed a compression of 20, 40, or 50% [32,48,68], and with a relaxation time between compressions ranging from 2 s to 15 s [32,57], most frequently 5 s [48,52,67]. Although an official method recommended for white and light wholegrain bread was issued by AACC (method 74–10.02 measurement of bread firmness-compression test), it was prone to variations due to the versatility of bread samples, which consequently hampers the results’ comparison. Similarly, the discrepancies in conditions applied during TPA of GFB samples are reflected in the results and impede real representation of the effects caused by the addition of antioxidants and accompanying compounds.

Among the textural parameters, hardness is considered the paramount GFB textural characteristic associated with consumers’ perception, referring to the bread freshness. GF cereals’ usage as an antioxidant source in GFB formulations resulted in a wide range of hardness values, 0.5–3957 N [57,60], while, for pseudocereals, the enveloped range was 0.2–41 N [57,62] (Table 4). The observed hardness for fruit and fruit by-products’ inclusion was in the range of 0.42–24.40 N [52,70] and, for tree fruits and leaves, ranged from 1.42 to 28.9 N [72,73] (Table 4). Regarding microalgae and algae inclusion, the reported hardness values were from 1.68 to 46 N [47,76] while, for by-products’ incorporation, the obtained hardness was 1.79 and 13.8 N [48,79] (Table 4). Generally, the hardness of GFB was reduced with the addition of GF cereals, namely wholegrain sorghum and millet [50,60], acerola fruit powder [65], strawberry seeds powder [67], extruded sour cherry pomace [68], pomegranate seed powder [70], and rosehip powder [52] from the group of fruit and fruit by-products, as well as Hemp inflorescence [38] and ground green coffee parchment [79]. Conversely, pseudocereals’ inclusion in GFB formulations, namely wholegrain buckwheat, buckwheat and chia flour blends, and buckwheat hulls, predominantly resulted in an increased hardness [43,45,61], and the same trend was noted with the addition of acorn flour [40,72], acorn and chickpea flour mixtures [33], and chestnut flour [73], as well as microalgae [75,76]. Furthermore, a negative correlation was constituted between the hardness and specific volume of the antioxidant-enriched GFB, regardless of the antioxidant compounds’ source, where increased crumb hardness resulted in a reduced GFB volume and vice versa [38,43,45,47,61,65,68,73,75]. The above-reported positive and negative influences upon antioxidant compounds’ addition on GFB crumb hardness is not yet clearly elucidated since they enter the formulation accompanied by dietary fibers, proteins, and other compounds which, given synergistic effects with water, usually contribute to a greater extent to the hardness perception. Here as well, the inclusion level of the antioxidant and accompanying compounds, as well as their type (such as soluble or insoluble dietary fibers), plays an important role in terms of crumb hardness [38,49,50,67]. The underlying action of different dietary fiber types in this respect is explained elsewhere [161].

Springiness, associated with the restoring ability upon deforming force retrieval after defined recovery time, decreased in GFB with wholegrain millet and wholegrain millet extruded flour [60] as well as chia flour and chia seed by-products [61,62], and remained unchanged with wholegrain red sorghum flour [50] and buckwheat hull [45] incorporation. Considering fruit and fruit by-products, the addition of acerola, pomegranate seed, and rosehip powder increased springiness values [52,65,70], while no changes were denoted with the inclusion of defatted blackcurrant and strawberry seeds [67], likewise for extruded sour cherry pomace [68]. Moreover, a rise in springiness was observed in GFB with acorn flour [40] and carob fiber [47], as well as that with broccoli leaf powder [48] and ground green coffee parchment [79].

The GFB cohesiveness was found to increase [60] but also remain unchanged [50,57] depending on the cereal used while, for pseudocereals and tree fruits, only a decrease in value was observed [40,45,57,61,73]. Chewiness represents a parameter that is tightly related to crumb hardness perception and, in most cases, exhibited the same tendencies in antioxidant-enriched GFB [40,45,50,73,77]. Resilience, accounted for as the instant crumb recovery upon compression, remained constant upon the addition of wholegrain red sorghum flour [50], buckwheat hulls [45], defatted blackcurrant and strawberry seeds [67], broccoli leaf powder [48], and ground green coffee parchment [79], but decreased with the inclusion of wholegrain millet and wholegrain millet extruded flour [60], chia flour [61], and acorn flour [40].

### 6.4. Effect of Antioxidant Compounds Source and Addition on Gluten-Free Bread Storage and Shelf-Life

Due to gluten absence and the application of a high percentage of starch in GFB formulations, the final GFB is prone to accelerated staling [162]. Alterations in the antioxidant content and activity in the enriched GFB potentially occurring during the storage have not been studied so far. Nevertheless, this should be examined in more detail in future research. Changes occurring during the storage of formulated GFB were primarily monitored through TPA in a time span from 1 to 7 days [43,45], but most frequently from 1 to 3 days [48,62,68,70,73,79].

Commonly, texture properties were negatively affected by storage and, with a prolonged storage time, the changes were more pronounced [45,48]. Crumb hardness was found to increase upon storage with the addition of wholegrain buckwheat flour [43] and coarse and fine buckwheat hulls for 7 days [45], as well as for chestnut flour [73], broccoli leaf powder [48], and ground green coffee parchment [79] after 3 days of storage compared to control GFB. Conversely, the addition of extruded sour cherry pomace resulted in hardness values lower than the prepared control GFB, which was attributed to the accompanied dietary fibers and their ability to bind several-times-higher water amounts compared to their mass [68]. Following the trend of hardness increase, the chewiness also increased with the storage of GFB with coarse and fine buckwheat hulls [45] and chestnut flour [73], but decreased with extruded sour cherry pomace incorporation [68]. The cohesiveness of the antioxidant compound-enriched GFB after storage in most cases decreased [45,48,73,79], as well as the resilience [48,73,79], while, regarding springiness, diverse influence was noted [48,68,70,79].

Considering that GFB requires more fat in its formulation compared to wheat counterparts accompanied by lower product humidity, lipid oxidation is regarded as one of the limiting factors for the product’s shelf-life. As a result of lipid oxidation, the acceptability and nutritional quality of the GFB are compromised due to off-odor and off-flavor compound formation as well as essential fatty acids’ content reduction [163]. The presence of antioxidant compounds in GFB aims to suppress the corresponding oxidation occurrence. The evaluation of oxidative stability is nowadays performed through product subjection to accelerated oxidative stress induced by increasing oxidative factors such as oxygen pressure and temperature under controlled conditions. Such analysis, so far, was performed in only one study with antioxidant-enriched GFB by using the instrument Oxitest (Velp Scientifica, Usmate Velate (MB), Italy), where 30 g of a minced bread sample was exposed to 90 °C and an oxygen pressure of 6 bar [79]. The exerted influence of the antioxidant compounds from ground green coffee parchment included in GFB was evident, as nearly 50%-higher oxidative stability was detected compared to control GFB, confirming the corresponding compounds’ ability to preserve the product during storage [79]. In the future, there is certainly a need for this kind of analysis, especially in products enriched with antioxidant compounds, where their actual effect on product quality and shelf-life can be assessed.

## 7. Sensory Properties of Antioxidant Compounds-Enriched Gluten-Free Bread

Apart from visual appeal, an acceptable sensorial perception to consumers represents another requirement that GFB should fulfill to reach the market. Depending on the chosen sensory methodology, the sensory evaluation of antioxidant-enriched GFB samples was most frequently assessed by a sensory panel with up to 10 panelists [34,54,67] or a population enveloping approximately 30, 50, or 70 respondents [35,38,45,49]. Nevertheless, a larger population of more than a hundred respondents was also engaged in studies for corresponding product evaluation (116 and 150 respondents) [43,71].

The population structures were comprised of respondents representing both genders and a wide age range (16–73 years), while half of the studies dealing with this topic disregarded reporting data on the population structure. Still, there is a noticeable predominance in terms of the number of female respondents who participated in the assessment, particularly when small groups of up to 10 members were evaluators (expert panelists) [34,42]. Larger population groups were mostly balanced regarding the number of male and female participants [49,62,71].

Considering the age range, the population involved in sensory evaluation was commonly comprised of all age groups from the young population (15–24 years), early adulthood (22–34 years), and early middle age (35–44 years) to late middle age (45–64 years) [38,43,75,79].

Respondents involved in the sensory analysis were randomly recruited through posters and e-mails [43], students and staff at the organization conducting research [72], habitual consumers [62], and even celiac patients [164]. Additionally, respondents were also trained or semi-trained for their role [54]; however, this was applicable only for small groups of respondents (6–15 panelists). Hence, the most frequent approach was the inclusion of untrained respondents [38,43,49,50,71,72,77,164]. Expert panelists were usually devoted to the characterization of quality descriptors (quantitative descriptive analysis, QDA) that were further used to facilitate product assessment by a larger population of respondents [42,71].

Antioxidant-enriched GFB samples were evaluated the same day they were produced, but more often 24 h after production [34,43,54,71]. The sample preparation for sensory assessment was comprised of GFB slicing (thickness 1–1.5 cm) [35,42,62,65] and random coding by a three digit number followed by a randomized sample supply to respondents. The sensory assessment of samples was usually performed in individual booths [43] under standard conditions enveloping normal lighting and room temperature (25 °C) as well as rinsing water to minimize residual effects between samples in compliance with adequate standards, although many studies omitted reporting on precise conditions during samples’ assessment.

To date, the consumer acceptance test applicable to a larger population of untrained responders was most frequently used for the antioxidant-enriched GFB evaluation following the nine-point hedonic scale method (1: dislike extremely, 5: neither like nor dislike, and 9: like extremely) [43,49,50,62,71,164]. However, some studies relied on seven-point [56,78] and five-point [35,72,75,76] hedonic scales for GFB assessment. The overall acceptability of antioxidant-enriched GFB followed by its quality attributes such as appearance, color, smell, aroma/flavor, taste, and texture [43,49,54] were generally judged by the respondents.

Furthermore, QDA represents another used approach for antioxidant-enriched GFB’s sensory characteristic measurement as estimated by a trained expert panel [34,42]. QDA, as well as the point system, precedes consumer acceptance tests and was applied in a limited number of studies on the corresponding product [42,71].

Additionally, the buying intention of respondents was parallel tested by the five-point hedonic scale in a certain number of recent studies [43,72,76].

According to the respondents, texture, taste, and appearance, followed by color and aroma, were the most affected by the antioxidant source inclusion in the GFB formulation [34,35,42,45,54,71,75,164].

The bitterness and astringency of many foods are associated with phenolic compounds’ presence; hence, the alteration of the antioxidant-enriched GFB’s taste was expected. Phenolic compounds of lower molecular weight such as phenolic acids, quercetin, and catechin are affiliated with a bitter taste while higher-molecular-weight phenolic compounds like tannins are more likely to result in an astringent taste [165]. Phenolic acids (free and bound form) were determined in cereals such as amaranth, purple maize, black and red rice [58], and pseudocereals (chickpea, quinoa, and buckwheat) [33,39]. Quercetin occurrence is most common in immature fruits [165] but it is also established in cereals (black rice and purple maize) alongside rutin [58] as well as in pseudocereals (buckwheat and quinoa) together with catechin [32,110] and rutin [43]. Tannins, however, besides fruits, are present in cereals (sorghum and millet), legumes, and diverse forage plants [165]. Since mentioned plant-based materials were used as antioxidant sources in GFB production (Table 1), the corresponding phenolic compounds’ presence imparted taste to GFB depending on the extent of inclusion. Nevertheless, the antioxidant source also plays an important role in the inclusion limit setting. Accordingly, a dry texture and uncharacteristic flavor of the GFB based on refined buckwheat flour substituted by 30 and 45% with wholegrain buckwheat flour were reported by many respondents [43]. In addition, the domination of the characteristic buckwheat aroma (basic flour) over the pleasant taste of fried onion (antioxidant source, 5%) was reported alongside enhancement in GFB’s texture and appearance [35]. Furthermore, a bitter note was prominent, especially in GFB based on purple corn flour, corresponding to its high content of rutin and quercetin; however, the effect on texture and appearance was not examined [58]. Considering fruit, the addition of 4 and 5% acerola fruit powder was reflected in a slightly sour taste of the GFB [65], as was the 10% inclusion of pomegranate seed powder [70]. A bitter aftertaste upon swallowing was also noted in GFB containing 5% hemp inflorescence [38] and higher than 3% carob flour [47], while a fishy flavor was reported by respondents after consumption of GFB containing 4% of the microalgae *T. chuii* [75]. Conversely, GFB containing 30% okara was characterized as very pleasant regarding taste, odor, chewiness, shape, cross-section structure, and other properties [71]. Unfortunately, conducted studies on antioxidant-enriched GFB so far have been scarcely devoted to the establishment of the relation between plant-based antioxidant compounds and taste, nor do they delve into the elucidation of potential mechanisms of action.

A more appealing appearance compared to control GFB was observed in most of the studies on antioxidant compounds’ inclusion [35,42,54,70,71,72]. Nevertheless, in most cases, the corresponding improvements were limited by maximal tolerable addition values depending on the additive origin, while a further increase in formulation share led to deterioration [56]. In addition, some studies reported lower scores regarding appearance and GFB overall acceptability compared to the control sample with the antioxidant compounds inclusion [45,62,77].

## 8. Bioaccessibility and Bioavailability of Antioxidant Compounds from Enriched Gluten-Free Bread

Knowing the proximate composition of formulated antioxidant-enriched GFB is insufficient to predict its beneficial health effects. Familiarity with the bioavailability and bioaccessibility of plant-based antioxidant compounds from GFB is of utmost importance for confirming the advocating assumptions related to their potential health benefits. To reveal the corresponding information, in vitro and in vivo studies (animal and human) can be applied. Although the most accurate results regarding the bioaccessibility and bioavailability of bioactive compounds are those involving human volunteers, in vitro studies are preferred, considering the possibilities of studying the effects of several endogenous or exogenous factors on bioaccessibility and bioavailability as well as minimizing the ethical, economical, and experimental constraints [166]. Nevertheless, in vitro and especially in vivo studies on antioxidant-enriched GFB are very scarce [35,36,47,78], leaving the actual functionality of the created product in the domain of assumption.

The in vitro method applied for antioxidant-enriched GFB simulates the conditions through three phases of digestion, namely oral (mouth), gastric (stomach), duodenal (small intestine) [47,78], and, in only one study, the fourth phase, i.e., colonic fermentation (large intestine) [36].

The size of the ground sample employed in the corresponding analysis is usually 1 g or 2 g [36,47,78]. The short oral digestion phase (5–10 min) takes place at pH 6.75 and includes the addition of simulated saliva fluid containing α-amylase from human saliva, mimicking chewing by a homogenizer [47,78]. For the following gastric phase, the pH is adjusted to 2 and inorganic salts along with porcine gastric mucosa pepsin are introduced and reacted with the sample under shaking for 60–120 min [36,47,78]. In the duodenal phase, upon pH adjustment to 7, pancreatin and bile extract were included and digestion was conducted in the dark for 120–150 min [47,78]. The obtained chyme is subjected to centrifugation and the supernatant is further used for the antioxidant compounds’ bioaccessibility assessment and also for intestinal absorption examination by performing dialysis (membrane cut-off 10–12 kDa) for 2 to 3 h [36,47]. The solution which passes the membrane (dialysate) is considered as the amount of antioxidant compound readily absorbed in the small intestine and addresses its bioavailability [36,47,167]. The gastric and duodenal phases of in vitro digestion take place under aerobic conditions and a temperature of 37 °C [47,78,166]. Additionally, the digestive process can be continued with the remaining non-dialyzable fraction’s subjection to simulated colonic fermentation by the addition of inoculum prepared with mouse fecal matter, which consequently passes another dialysis (pH 7.5, agitation at 40 °C in the dark for 24 h, anaerobic conditions) [36].

The resulting dialysates from the duodenal and colonic fermentation phases were most frequently subjected to determination of the TPC and ABTS [36,47,78] as well as, to a lesser extent, FRAP, CHEL, and RED [36,47]. It was observed that the TPC content increased after in vitro digestion of GFB with carob fiber [47], coffee silverskin, and coffee husk extract [78] compared to results before digestion. Conversely, opposite results were reported for GFB enriched with whole and polished white sorghum flour, where a reduction in the TPC was noticed in dialysates from duodenal and colonic fermentation [36]. The ABTS values were also increased after the inclusion of coffee silverskin and coffee husk extract [78] and whole and polished white sorghum flour [36] in GFB, while, with the carob fiber addition after the initial decrease, the same trend was observed with 4 and 5% inclusion [47]. Hence, the influence of the antioxidant addition level was also notable, since the TPC decreased with carob fibers’ addition, while the ABTS was found to increase [47].

Characteristics such as the molecular mass, chemical structure, and addition level in product, as well as food matrix and digestion pathways, positively or negatively affect the bioaccessibility and bioavailability of bioactive compounds [168]. The discrepancies in the TPC content resulting from classical chemical extraction and in vitro digestibility can also be attributed to various used organic solvents which do not entirely resemble human digestion [36]. Furthermore, the limitations regarding in vitro antioxidant capacity assays for the estimation of health beneficial effects of antioxidant-enriched GFB are associated with changes in the antioxidant activity of polyphenols as a result of passed digestion and the variable extent of bioavailability among diverse polyphenol classes with the already mentioned lack of standardized methods [39]. All of this leads to unreliable results which are hardly comparable between already scarce corresponding studies.

The in vivo methods applied on GFB enriched with red onion peel [35] were free oxygen radicals (FORT) and free oxygen radicals defense-in vivo antioxidant activity (FORD) assays, which were used as oxidative stress markers [169]. Details on the basic principles of their action can be found in Bedrníček et al. [35]. The corresponding assays were performed with a specialized kit which requires small amounts of capillary blood taken prior and 90 min upon enriched GFB consumption (20 µL for FORT and 50 µL for FORD), as well as absorbance measurement at 505 nm [35]. In the FORT assay, the results are expressed as mmol H_2_O_2_ eq/L blood while, in the FORD assay, the results are delivered as mmol TE/l blood. A double increase in the FORD values was reported by the authors upon administration of 200 g GFB enriched with onion peel containing 68.02 mg of flavonols (~65 mg QE) compared to the same portion of control bread while, regarding FORT, no significant changes were detected [35]. Nevertheless, the authors emphasized the absence of statistically significant changes for both assays 90 min after enriched GFB consumption and attributed them to an insufficient amount of QE or the experiment length, highlighting the need for further research [35].

## 9. Summary and Conclusive Remarks

Reaching a proper balance in the oxidative-reduction system is needed for health maintenance. For celiac patients, the fulfillment of this task is of paramount importance considering that greater exposure to ROS leads to further deterioration of the mucosa. Plant-based antioxidant-rich raw materials and additives (GF cereal, pseudocereal, and legume flours, agricultural and food industry by-products, and extracts thereof) coupled with applied pre-treatments and technologies (germination, sourdough, extrusion, 3D printing) are promising strategies for GFB enrichment with antioxidants. However, perceived constraints in results’ expression due to diverse sample preparation techniques, standards, and assays used hamper the possibility of meaningful comparison of the mentioned strategies’ effectiveness and impose a necessity of corresponding methods’ standardization. Still, the extent of GFB antioxidant enhancement was primarily dependent on the plant-based additive’s origin, its richness in antioxidant compounds, the amount added, and changes in antioxidant compounds during bread-making.

Apart from antioxidant enrichment, in most cases, the GFB technological quality and sensory acceptability in terms of specific volume, crumb softness, color, aroma, and taste were improved and more appealing. The extent of corresponding improvements was limited to the antioxidant source and the amount added but also to the involvement of accompanying dietary fibers and other structuring ingredients present in the GFB formulation. Despite much information having been gathered, this literature review reveals the existence of many gaps in the topic. Scarce are studies devoted to the exploration of alterations in antioxidant compounds during bread-making and GFB storage, as well as those disclosing the relationship between plant-based antioxidant compounds and GFB volume, texture, and taste. Studies on the bioaccessibility and bioavailability of antioxidant compounds from enriched GFB are also lacking; however, those conducted reveal both positive and negative effects, implying limitations regarding antioxidant determination assays in estimation and results comparison.

If we consider the research on antioxidant-enriched GFB as a target, outer target circles can envelope the mainly investigated antioxidant content and activity in enriched GFB, predominantly explored plant-based antioxidant sources and the amounts applied, and partially investigated effects on GFB technological and sensory properties. Inner target circles may consist of strategies for enhancing the antioxidant content in GFB and changes in antioxidant compounds during bread-making and storage, with their bioaccessibility and bioavailability in the target center, which requires further, deeper investigation to elucidate the potential mechanisms of action and confirm the advocating assumptions related to their health benefits.

## Figures and Tables

**Table 1:** Summary of methods applied for quantification and identification of antioxidant compounds in gluten-free bread together with antioxidant source and addition level applied.

Basic Flour	Antioxidant Source	Addition Level (%)	Quantification Methods	Profiling	Reference
Gluten-free cereals, pseudocereals and legumes					
Rice flour, potato starch	Amaranth, quinoa, buckwheat, sprouted buckwheat	50 100	TPC, DPPH, FRAP	Simple polyphenols Flavonoids HPLC–DAD	[39]
/	Soaked and germinated brown rice flour	100	TPC, ORAC	/	[44]
/	Quinoa flour, wholegrain sorghum flour, brown millet flour, wholegrain rice flour	100	TPC, DPPH, ABTS, FRAP	/	[55]
Rice flour	Buckwheat hulls	3.61, 3.83 7.23, 7.66	TPC, DPPH, ABTS, ORAC, FRAP, QUENCHER	/	[45]
Refined buckwheat flour	Wholegrain buckwheat flour	30, 45	TPC, ORAC	Rutin and quercetin HPLC–DAD	[43]
Rice flour	Buckwheat flour (light and wholegrain)	10, 20, 30	TPC, antioxidant activity with β carotene, RED, DPPH, CHEL	Rutin and quercetin HPLC–DAD	[46]
Maize starch	Dehulled buckwheat flour	10, 20, 30, 40	TPC, DPPH, ABTS, reducing capacity by voltammetry method	/	[42]
Rice flour and maize starch	Buckwheat, quinoa and amaranth flour	15, 30, 45	TPC, DPPH	/	[56]
Maize starch and potato starch	Maize flour, buckwheat flour and amaranth flour	10	TPC, TFC, ABTS, phenolic acids, flavonols, anthocyanins, tannins	/	[57]
/	Rice flour (white, brown, black, red and wild) Corn flour (purple and yellow)	100	/	Anthocyanin Flavonoids Phenolic acids RP-UHPLC-ESI-MS	[58]
Maize starch and potato starch	Wholegrain red sorghum flour	10, 20, 30, 40	TPC DPPH, ABTS, FRAP, CUPRAC	/	[50]
Sorghum flour and maize starch	Germinated white sorghum flour	20	TPC, TFC, DPPH tannins	/	[54]
Potato starch and cassava flour	Sorghum (white, brown and bronze)	22.26	ORAC	/	[59]
Rice flour and cassava flour	White sorghum flour	85	TPC, ABTS, FRAP	Phenolic acids Flavonoids HPLC-DAD-ESI-MS/MS	[36]
/	Wholegrain millet and wholegrain millet extruded flour	100, 50	TPC, DPPH, FRAP	/	[60]
Buckwheat flour (light and wholegrain)	Chia flour	9.8, 10	DPPH	/	[61]
Buckwheat flour (common and Tartary)	Chia flour	10	TPC, TFC, FRAP, ORAC	/	[37]
Maize and rice flour	Chia and chia seed residues	5	TPC, DPPH	/	[62]
Maize and quinoa flour	Germinated sweet lupin and fenugreek mixtures	5, 10, 15, 20	DPPH, ABTS, FRAP TFC, total phenolic acids	/	[63]
Maize flour	Extruded lentil flour	15	TPC, DPPH, ORAC, FRAP, ABTS, QUENCHER	/	[41]
Wholegrain rice, wholegrain millet flour, corn starch, corn extrudate	Yellow pea flour	25	TPC, DPPH, FRAP	Phenolic acids HPLC–DAD	[64]
Fruit and fruit by-products					
White rice	Acerola fruit powder	1, 2, 3, 4, 5	TPC, DPPH, ABTS, RED	/	[65]
Maize starch and potato starch	Apple pomace	5, 10, 15	TPC, TFC ABTS	Flavonols Phenolic acids Flavon-3-ols Dihydrochalcones UPLC-PDA-MS/MS	[66]
Maize starch and potato starch	Defatted blackcurrant and strawberry seeds	5, 10, 15	TPC, ABTS	/	[67]
Rice flour, maize starch and potato starch	Extruded sour cherry pomace and rice flour	10	TPC, TFC, ABTS, phenolic acids, anthocyanins	/	[68]
Gluten-free flour mixture	Grape seed flour	3, 6, 9	TPC, FRAP	/	[69]
Rice and field bean semolina	Pomegranate seed powder	2.5, 5, 7.5, 10	TPC, DPPH, ABTS, RED, •OH	/	[70]
Gluten-free flour	Rosehip powder, rosehip encapsulate	7	TPC, DPPH, total carotenoids	/	[52]
Vegetables and vegetable by-products					
Maize starch and potato starch	Freeze-dried red and purple potatoes	5	TPC, TFC, DPPH, ABTS, FOMO flavonols, phenolic acid, anthocyanins, carotenoids	Chlorogenic acid Neo-chlorogenic acid Rutin Quercetin HPLC	[53]
Maize starch and potato starch	Red and purple potato pulp	5, 7.5, 10	TPC, TFC, flavonols, anthocyanins ABTS	/	[51]
Maize starch and potato starch	Broccoli leaf powder	5	TPC ABTS, DPPH, PCL	/	[48]
Unhusked white buckwheat flour, corn flour, rice flour, linseed flour	Fried red onion, dried red onion and red onion peel	5	TPC, DPPH, FRAP FORD, FORT (in vivo)	Quercetin Quercetin-3,4′-O-diglucoside Quercetin-4′-O-glucoside Rutin HPLC-MS/MS	[35]
Buckwheat flour, rice flour and millet flour	Okara	10, 20, 30	TPC, DPPH, FRAP, ABTS	/	[71]
Herbs					
Rice flour	Hemp inflorescence	1, 2, 3, 4, 5	TPC, TFC, DPPH, FRAP	/	[38]
Tree fruits and leaves					
Rice flour	Acorn flour	23, 35	TPC, DPPH, FRAP, ABTS	/	[40]
Buckwheat flour, rice flour and potato starch	Acorn flour	23, 35	TPC, TFC, ODC, DPPH, FRAP, ABTS	Phenolic acids Flavonoids RP-HPLC-DAD	[72]
Rice flour and maize starch	Acorn and chickpea flour mixtures	30	TPC, TFC, DPPH, ABTS, FRAP	Phenolic acids HPLC-DAD Tocopherols and carotenoids HPLC-FLD	[33]
Maize starch and potato starch	Chestnut flour	10, 20	DPPH	/	[73]
White rice, maize, and buckwheat flour	Carob fiber (commercial)	1, 2, 3, 4, 5	TPC, ABTS, CHEL, FRAP	/	[47]
Rice flour	Kefir beverage with carob leaves	155 mL water replaced with 150 mL kefir	DPPH, ABTS	/	[74]
Rice and field bean semolina	*Moringa oleifera* leaves powder	2.5, 5, 7.5, 10	TPC, DPPH, ABTS, RED, •OH	/	[49]
Microalgae and algae					
Buckwheat flour, rice flour and potato starch	Microalgae *Tetraselmis chuii*	1, 2, 4	TPC, DPPH, FRAP	/	[75]
Buckwheat flour, rice flour, potato starch	Microalgae biomass and ethanol-treated microalgae biomass	4	TPC, DPPH, carotenoids, chlorophyll a and chlorophyll b	/	[76]
White rice flour, maize flour and millet flour	Brown algae powder	2, 4, 6, 8, 10	TPC, ABTS, CHEL, FRAP, •OH	/	[77]
Food industry by-products					
Maize starch, inulin and rice protein	Coffee silverskin and coffee husk extract	2.5	TPC, ABTS, QUENCHER	/	[78]
Maize starch and rice flour	Ground green coffee parchment	2	TPC, DPPH	/	[79]
Maize starch and potato starch	Flaxseed oil cake extract	25, 50, 75, 100	TPC ABTS, DPPH, FRAP, PCL	/	[34]

TPC, total phenolic content; TFC, total flavonoid content; DPPH, 2,2-Diphenyl-1-picrylhydrazyl assay; ABTS, 2,2-Azinobis 3-ethylbenzthiazoline-6-sulfonic acid radical scavenging assay; FRAP, ferric reducing antioxidant power assay; PCL, photochemiluminescence assay; •OH, Hydroxyl radical scavenging assay; CHEL, chelating activity; ODC, ortho-diphenols content; FORD, free oxygen radical defense assay; FORT, free oxygen radical assay; FOMO, phosphomolybdenum complex methods; RED, reducing power; ORAC, oxygen radical absorbance capacity; CUPRAC, copper reducing antioxidant capacity; HPLC-DAD, High-Performance Liquid Chromatography-Diode Array Detector; RP-UHPLC-ESI-MS, Reversed-phase-ultra-high-performance liquid chromatography-electrospray ionization-mass spectrometry; HPLC-DAD-ESI-MS/MS, High-Performance Liquid Chromatography-Diode Array Detector-ESI source-tandem Mass Spectrometry; UPLC-PDA-MS/MS, Ultra-Performance Liquid Chromatography-tandem Mass Spectrometry; HPLC-MS/MS, High-Performance Liquid Chromatography-tandem Mass Spectrometry; RP-HPLC-DAD, Reverse Phase-High-Performance Liquid Chromatography-Diode Array Detector; HPLC-FLD, High-Performance Liquid Chromatography-Fluorescence detector.

**Table 4:** Specific volume and crumb hardness values reported for gluten-free breads enriched with plant-based antioxidant compounds.

Antioxidant Source	Addition Level (g/100 g%)	Volume (cm^3^)	Specific Volume (cm^3^/g)	Crumb Hardness (N)	Reference
Gluten-free cereals, pseudocereals and and legumes					
Pregelatinized rice	100	/	0.6 ± 0.1 *	/	[32]
Wholegrain rice flour	100	/	1.81	13.74 ± 0.23	[55]
Maize flour	10	548.6 ± 10.5 **	2.87 ± 0.02	~0.5	[57]
Broccoli leaf powder	5	/	3.08 ± 0.16	13.80 ± 0.07	[48]
Millet	100	/	1.3 ± 0.0 *	/	[32]
Brown millet flour	100	/	1.64	25.70 ± 0.15	[55]
Wholegrain millet and wholegrain millet extruded flour	100, 50	0.59–0.93	/	623.6–3957	[60]
Wholegrain sorghum flour	100	/	1.52	21.47 ± 0.32	[55]
Wholegrain red sorghum flour	10, 20, 30, 40	/	2.41–3.21	6.90–13.92	[50]
White sorghum flour	85	/	4.68–4.78	14.87–25.87	[36]
Quinoa flour	100	/	1.92	10.81 ± 0.14	[55]
Buckwheat	100	/	2.0 ± 0.1 *	/	[32]
Buckwheat hulls	3,6	396–470 **	2.5–2.8	2.2–3.2	[45]
Wholegrain buckwheat flour	30, 45	/	2.88–2.93	30.34–33.87	[43]
Buckwheat flour	10	35.5 ± 6.1 **	3.12 ± 0.01	~0.3	[57]
Amaranth flour	10	563.3 ± 3.7 **	2.78 ± 0.03	~0.2	[57]
Chia flour	9.8, 10	/	1.73–2.04	8.54–12.65	[61]
	10	397–428	1.34–1.49	/	[37]
Chia and chia seed waste	5	~115–130	/	~37–41	[62]
Fruit and fruit by-products					
Acerola fruit powder	1, 2, 3, 4, 5	502–571.6	/	9.9–11.5	[65]
Defatted blackcurrant seeds	5, 10, 15	485–527	/	~1.9–3.7	[67]
Defatted strawberry seeds	5, 10, 15	575–608	/	~1.7–2.1	[67]
Extruded sour cherry pomace and rice flour	10	521–562 **	/	~0.6–0.9	[68]
Pomegranate seed powder	2.5, 5, 7.5, 10	/	~2.65–2.85	20.03–24.40	[70]
Rosehip powder, rosehip encapsulate	7	/	/	0.42–1.85	[52]
Herbs					
Hemp inflorescence	1, 2, 3, 4, 5		~0.98–1.08	13.11–15.76	[38]
Tree fruits and leaves					
Acorn flour	23, 35	/	/	4.24–5.07	[40]
		965.56–1255	/	22.74–28.90	[72]
Acorn and chickpea flour mixtures	30	/	1.85–3.27	1.80–7.10	[33]
Chestnut flour	10, 20	/	2.6–3.1	1.42–4.04	[73]
Carob fiber (commercial)	1, 2, 3, 4, 5	~157–165	/	~25–28.5	[47]
*Moringa oleifera* leaves powder	2.5, 5, 7.5, 10	/	~2.37–2.48	22.40–26.04	[49]
Microalgae and algae					
Microalgae *Tetraselmis chuii*	1, 2, 4	612–642	/	3.74–6.28	[75]
Microalgae biomass and ethanol-treated microalgae biomass	4	/	1.95–2.96	1.68–4.85	[76]
Brown algae powder	2, 4, 6, 8, 10	~136–140.5	/	~32–46	[77]
Food industry by-products					
Ground green coffee parchment	2	/	~3.65	1.79 ± 0.35	[79]
Flaxseed oil cake extract	25, 50, 75, 100	/	2.39–3.06	/	[34]

* measured by Volumetric Analyzer (Perten Instruments); ** measured by Volscan Profiler (Stable Micro Systems, Godalming, UK); ~values taken from graphical representations of the reported results.

## Data Availability

Data is contained within the article.
